# Targeted mutagenesis of a conserved anther‐expressed P450 gene confers male sterility in monocots

**DOI:** 10.1111/pbi.12633

**Published:** 2016-10-14

**Authors:** A. Mark Cigan, Manjit Singh, Geoffrey Benn, Lanie Feigenbutz, Manish Kumar, Myeong‐Je Cho, Sergei Svitashev, Joshua Young

**Affiliations:** ^1^ Trait Technologies, DuPont Pioneer Johnston IA USA; ^2^ Trait Technologies, DuPont Pioneer Hayward CA USA; ^3^ Present address: Department of Plant Biology University of California One Shields Avenue Davis CA USA

**Keywords:** endonuclease, male sterile, rice, sorghum, wheat

## Abstract

Targeted mutagenesis using programmable DNA endonucleases has broad applications for studying gene function *in planta* and developing approaches to improve crop yields. Recently, a genetic method that eliminates the need to emasculate the female inbred during hybrid seed production, referred to as Seed Production Technology, has been described. The foundation of this genetic system relied on classical methods to identify genes critical to anther and pollen development. One of these genes is a P450 gene which is expressed in the tapetum of anthers. Homozygous recessive mutants in this gene render maize and rice plants male sterile. While this P450 in maize corresponds to the male fertility gene *Ms26*, male fertility mutants have not been isolated in other monocots such as sorghum and wheat. In this report, a custom designed homing endonuclease, Ems26+, was used to generate *in planta* mutations in the rice, sorghum and wheat orthologs of maize *Ms26*. Similar to maize, homozygous mutations in this P450 gene in rice and sorghum prevent pollen formation resulting in male sterile plants and fertility was restored in sorghum using a transformed copy of maize *Ms26*. In contrast, allohexaploid wheat plants that carry similar homozygous nuclear mutations in only one, but not all three, of their single genomes were male fertile. Targeted mutagenesis and subsequent characterization of male fertility genes in sorghum and wheat is an important step for capturing heterosis and improving crop yields through hybrid seed.

## Introduction

Crop improvement on a global scale is required to meet growing food demands (Parry and Hawkesford, 2012; Ray *et al*., 2013). The expansion of yield gains made by developing hybrid crops, rather than varieties, would be an important step to achieve such progress where harnessing the advantages of heterosis is the main objective of hybrid breeding (Duvick, 1999; Melchinger, 1999; Tester and Landrige, 2010). Genetic divergence between parent lines has been demonstrated to correlate with the extent to which heterosis of a hybrid increases. Hybrid seed production requires that a male parent inbred pollinates a genetically distinct female inbred parent that is either male sterile or has been emasculated. This is straightforward in monoecious plants such as maize that have separate male (tassel) and female (ear) flowers on the same plant, where detasseling can emasculate the female parent during hybrid seed production. While detasseling has been used for hybrid maize seed production, physical emasculation is not a scalable solution for crop species with perfect flowers such as rice, sorghum, barley, millet and wheat. Historically, cytoplasmic male sterility (CMS) has been used in these crops for hybrid seed production; however this method is not effective in all germplasm due to the dependence on nuclear restorer genes specific for a given cytoplasm (Dwivedi *et al*., 2008; Li and Yuan, 1999; Reddy *et al*., 2005; Whitford *et al*., 2013). Recently, a novel hybrid seed production system has been described for maize that is not limited by the genetic background (Wu *et al*., 2015). This Seed Production Technology (SPT) system involves restoring pollen formation while maintaining a homozygous nuclear recessive mutation in the maize male fertility gene *Ms45*. Although homozygous *ms45* maize plants are male sterile, pollen formation can be restored in plants that contain a transformed version of the *Ms45* gene. In the SPT system, only the *ms45* allele and not the Ms45 restoration cassette is transmitted through pollen. The resultant *ms45* progeny are used as females during hybrid production; thus, emasculation by detasseling is not necessary as these females are genetically male sterile. Although this system originally incorporated the maize *Ms45* gene, it is applicable to other crops and to other nuclear male sterile mutations, whether dominant or recessive.

Extending this hybrid seed production system to other monoecious crops could afford the ability to maximize yield potentials through heterosis despite important differences such as asynchronous flower development and architecture. This is particularly relevant for rice, sorghum and wheat. However, deployment of a genetically based hybrid seed production system in these crops requires the identification and isolation of male fertility genes. While male fertility genes and mutants have been described and isolated in maize (Cigan *et al*., 2001; Neuffer *et al*., 1997) and rice (Guo and Liu, 2012; Yamagata *et al*., 2007) by classical genetic approaches, there is a paucity of available mutants in crops such as sorghum and wheat. As opposed to the use of chemicals or irradiation to generate random mutations and the associated time‐consuming screening of mutagenized plants, recent progress in DNA double‐strand break (DSB) technologies holds great promise for the systematic generation of male sterility mutants in these agriculturally important crops. A variety of nucleases can be employed for the generation of targeted DSBs; zinc‐finger nucleases (ZFN), I‐*Cre*I‐based homing endonucleases (meganucleases), transcription activator‐like effector nucleases (TALEN) and the Cas9‐guide RNA system (often referred to as ‘CRISPR‐Cas’) have been used to produce mutations in various plant species (Kumar and Jain, 2015; Osakabe and Osakabe, 2015; Svitashev *et al*., 2015; Voytas and Gao, 2014). DSBs are repaired through endogenous cellular processes of nonhomologous end‐joining (NHEJ) or homology‐directed repair; the NHEJ repair pathway can often be inexact resulting in mutations at the site of the DSB and in this way targeted mutagenesis is achieved. Such a directed mutagenesis strategy utilizing a redesigned I‐*Cre*I homing endonuclease to target the *Ms26* male fertility gene in maize has recently been reported (Djukanovic *et al*., 2013). As demonstrated in rice, the rice *Ms26* ortholog encodes for a cytochrome P450 mono‐oxygenase enzyme (OsCYP704B2) and is expressed in the tapetum and microspores in the developing anther (Hobo *et al*., 2008; Li *et al*., 2010). The homozygous recessive *ms26* mutants in maize and rice are defective in both tapetum and microspore development resulting in male sterility (Li *et al*., 2010; Loukides *et al*., 1995). The redesigned endonuclease described by Djukanovic *et al*. (2013), Ems26+, targets a 22‐nt sequence found in the 5th exon of *Ms26*. DSB generated by Ems26+ within the predetermined chromosomal recognition site and NHEJ repair resulted in mutations in the *Ms26* gene and loss of function. Maize plants containing homozygous recessive *ms26* mutant alleles were phenotypically normal, producing tassels that contained developed spikelets, with the exception that anthers did not shed pollen. These mutant *ms26* alleles were transmitted to progeny at the expected segregation ratio (Djukanovic *et al*., 2013). The male sterile phenotype generated by targeted gene disruption using the Ems26+ endonuclease was indistinguishable from *ms26* maize mutants generated by transposon‐tagging (Loukides *et al*., 1995; Fox, T., DuPont Pioneer, unpublished observation).

This report demonstrates that the Ems26+ endonuclease can generate targeted mutations in the orthologs of *Ms26* genes of rice (*Oryza sativa*,* Os*), sorghum (*Sorghum bicolor*,* Sb*) and common wheat (*Triticum aestivum*,* Ta*). Orthologous *Ms26* mutations in rice and sorghum plants, such as maize (*Zea mays* L., *Zm*), confer a recessive male sterile phenotype, and restoration of fertility in these mutant sorghum plants was achieved by a transformed copy of maize *Ms26*. In addition, using a conditionally regulated Ems26+, mutations in the wheat *Ms26* ortholog were recovered in all three genomes of hexaploid spring wheat. These results demonstrate the utility of nuclease‐facilitated gene editing to produce male fertility gene mutants for the purpose of unlocking yield potential through exploiting heterosis in these major food crops.

## Results

**Figure 1:**
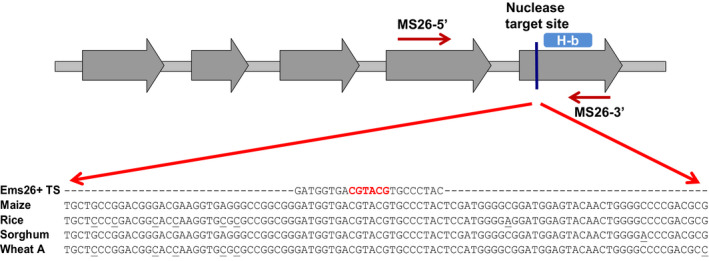
Ems26+ endonuclease target site is conserved across maize, rice, sorghum and wheat. Gene structure illustration of maize *Ms26*; exons (grey arrows) and the haem‐binding (H‐b) domain are shown. The haem‐binding domain resides in the 5th exon of the maize *Ms26* (Gene ID: 100191749) gene and sorghum (Gene ID: 8082128) ortholog. This domain is also found in the last exon of the rice (Gene ID: 4331756) and the wheat (Fielder A‐genome) *Ms26* orthologs, which each consists of four exons. The Ems26+ target site is depicted as a vertical line upstream of the H‐b domain. Sequence differences in rice, sorghum and wheat A‐genome as compared to the maize *Ms26* gene are indicated as underlined nucleotides. The UNIMS26 primer pair (arrows labelled MS26‐5′ and MS26‐3′) was used to amplify the region of interest from isolated genomic DNA to screen for *Ms26* mutations. A *Bsi* WI restriction enzyme site (red font) present in the 22‐nt Ems26+ target site allowed for *Ms26* mutation screening by determining resistance to *Bsi* WI digestion.

The maize *Ms26* gene is a member of a P450 protein family whose expression is limited to developing anthers (Cigan *et al*., 2005). Biochemical characterization indicated putative roles of the rice Ms26 ortholog (OsCYP704B2) catalysing ω‐hydroxylated C16 and C18 fatty acids as well as synthesis and transport of sporopollenin precursor components important to pollen wall formation (Li *et al*., 2010). Maize and rice plants carrying homozygous recessive mutations disrupting the coding‐frame of the *Ms26* and *OsCYP704B2* genes, respectively, resulted in an inability of these plants to generate fertile pollen grains (Djukanovic *et al*., 2013; Li *et al*., 2010). Consistent with the importance of this P450 for pollen development, protein sequences with high homology to the maize Ms26 were identified in sorghum and wheat by searching plant sequence databases. The sorghum and wheat *Ms26*‐like sequences along with the rice *OsCYP704B2* gene were, therefore, referred to herein as *SbMs26*,* TaMs26* and *OsMs26*, respectively. Although there is significant sequence identity across these crop species (Figure S1 and Text S1), nothing is known regarding the functional conservation of this P450 in sorghum and wheat. As shown in Figure 1, the 22‐nt Ems26+ recognition site is conserved across these monocots and resides in the last exon of each gene, and this exon contains the important haem‐binding loop common to haem‐thiolate cytochrome P450s (Werck‐Reichhart and Feyereisen, 2000). To determine whether the *Ms2*6 gene is required for male fertility, several directed mutagenesis approaches using the Ems26+ endonuclease were developed and tested in rice and then used to determine whether this gene is required for male fertility in sorghum and wheat.

### Ems26+‐generated mutations in rice

**Figure 2:**
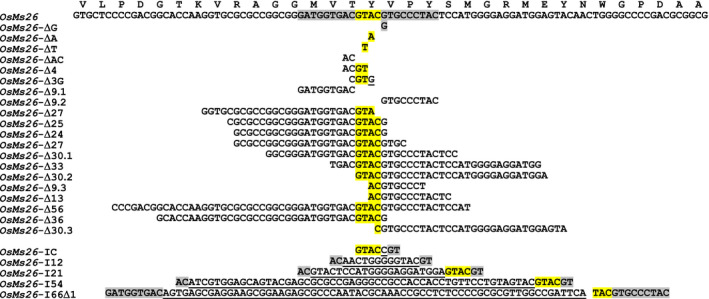
Ems26+‐generated *OsMs26* mutations in rice. Shown in the WT reference sequence, the 22‐nt Ems26+ recognition sequence is highlighted in grey and yellow. The latter indicates the 4 bp overhang, 5′‐GTAC‐3′, produced by nuclease cutting and is highlighted throughout for orientation purposes. For each mutant, deleted nucleotides are shown and inserted nucleotides are shown as underlined. Amino acid sequence corresponding to the *OsMs26* gene is provided as single letters above the WT nucleotide reference sequence.

Young rice callus [(*Oryza sativa* L. ssp. Japonica (cv. Kitaake)] initiated from germinating seed was used as transformation targets for biolistic‐ or *Agrobacterium*‐mediated delivery of vectors that contained the custom endonuclease, Ems26+. For biolistic transformation, two vectors were constructed: (i) a vector containing a codon‐optimized Ems26+ under the transcriptional regulation of the maize ubiquitin promoter (UBIpro:Ems26+) enabling constitutive expression, and (ii) a vector containing a herbicide resistance selectable marker phosphinothricin acetyltransferase (PAT) fused to the red fluorescence protein (RFP) gene and placed under the regulation of the maize END2 promoter. This vector allowed transformed rice cells to grow in the presence of the herbicide glufosinate while monitoring red fluorescence, when codelivered into 2‐week‐old, seed‐derived rice callus. Ems26+ contains a nuclear localization signal at the N‐terminus and an intron to eliminate expression in bacterial cells used for vector propagation (Djukanovic *et al*., 2013). Approximately 6 weeks after bombardment, callus sectors that grew on media containing glufosinate were screened for mutations at the 22‐nt Ms26 target site. Due to the presence of a *Bsi*WI restriction site (5′‐CGTACG‐3′) across the centre of the Ems26+ recognition sequence and at the site of Ems26+‐mediated DNA cleavage (Figure 1), PCR amplification and digestion by *Bsi*WI was used as an initial screening tool to detect *Ms26* mutations. PCR amplicons resistant to *Bsi*WI digestion would be indicative of sequence changes at this recognition site due to cutting by Ems26+ and imprecise repair by NHEJ (Djukanovic *et al*., 2013). Genomic DNA isolated from these glufosinate‐resistant callus sectors was amplified using the primer pair UNIMS26 5′‐2 and UNIMS26 3′‐1 to generate a 653‐bp amplicon that was then subjected to digestion with *Bsi*WI. Products of these reactions were electrophoresed on 1% agarose gels and screened; amplicons containing wild‐type sequence across the Ems26+ recognition site would produce 376‐ and 277‐bp DNA digestion products (Figure S2, lanes 2, 4). Screening of 292 glufosinate‐resistant events generated by cobombardment with the Ems26+ vector identified 22 callus sectors that contained PCR products resistant to *Bsi*WI digestion. Subcloning and DNA sequence analysis of these PCR products revealed a variety of mutations across the Ms26 target site, which included single‐ and multiple‐nucleotide insertions and deletions (Figure 2).

**Figure 3:**
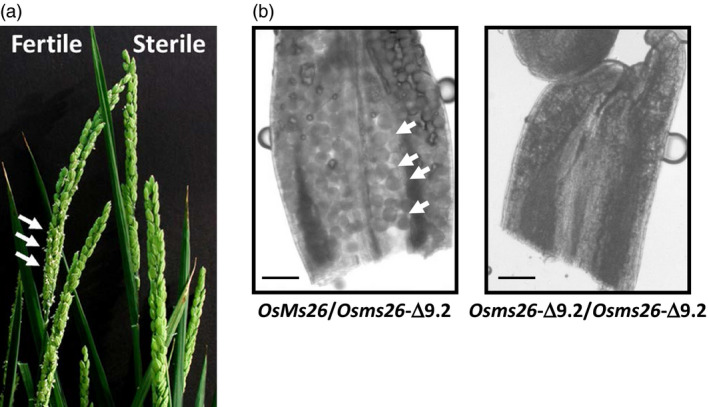
Male sterile rice generated from targeted mutagenesis of the *OsMs26* gene via bombardment delivery of Ems26+. (a) Rice panicles from male fertile (left; *OsMs26/Osms26*‐Δ36) and male sterile (right; *Osms26*‐Δ36/*Osms26*‐Δ36*)* plants. Dehisced anthers are shown with white arrows. (b) Longitudinal anther squash from heterozygous *OsMs26*/*Osms26‐*Δ9.2 male fertile plants (left) containing microspores (white arrows) and homozygous *Osms26‐*Δ9.2/*Osms26‐*Δ9.2 male sterile plants (right). Magnification bar = 100 μM.

To examine the male fertility phenotype of rice plants containing *OsMs26* mutations generated by Ems26+, plants were regenerated from glufosinate‐resistant callus sectors, analysed for *OsMs26* mutations and allowed to set self‐pollinated seed (T1 seed). T1 seed from three plants containing nonidentical *OsMs26* mutations, but lacking the randomly integrated transformation vectors, were advanced for progeny testing. Using the *Bsi*WI digestion assay, genomic DNA collected from progeny plantlets was amplified with the UNIMS26 primer pair and screened for mutations at the Ms26 target site. Heterozygous *OsMs26/Osms26* (Figure S2, lane 4) and homozygous *Osms26/Osms26* (Figure S2, lane 6) mutant plants were advanced and scored for their ability to form pollen by analysing seed set upon self‐fertilization. Throughout vegetative growth, these plants appeared phenotypically normal and flowered at the same time as wild‐type plants with the exception that dehisced anthers were not observed in the homozygous recessive *Osms26* plants (Figure 3a). Microscopic examination of anthers from *OsMs26/Osms26* plants staged at late uninucleate microspore development revealed normal appearing microspores, which continued to develop into mature, fertilization‐competent pollen. However, examination of anthers from *Osms26*/*Osms26* plants revealed that microspores arrested development immediately after tetrad release, whereupon these plants contained anthers devoid of mature pollen (Figure 3b). These observations were similar to those reported by Loukides *et al*. (1995) and Li *et al*. (2010). Surprisingly, as shown in Figure 3b, the in‐frame deletion of nine nucleotides in *Osms26*‐Δ9.2 conferred a male sterile phenotype. In summary, all *OsMs26/Osms26* plants were male fertile; and all *Osms26/Osms26* plants were male sterile (Table 1). In addition, homozygous *Osms26/Osms26* plants in this study were female fertile as demonstrated by their ability to set seed when fertilized with wild‐type rice pollen.

**Table 1:** Male fertility phenotypes of T1 progeny plants with heterozygous or homozygous *OsMs26* mutations

Mutation	Plants	*OsMs26*/*Osms26*		*Osms26*/*Osms26*	
		Male fertile	Male sterile	Male fertile	Male sterile
*Osms26*‐Δ9.2	18	10	0	0	8
*Osms26*‐Δ36	9	5	0	0	4
*Osms26*‐I66Δ1	15	11	0	0	4

### Inducible Ems26+ for producing double‐strand breaks

**Figure 4:**
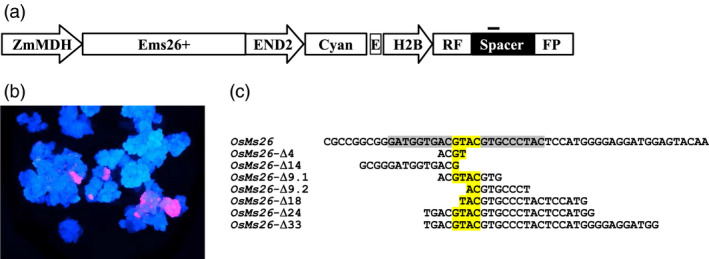
*OsMs26* mutations in rice generated using a stably integrated cassette containing Ems26+ under transcriptional control of a maize temperature‐regulated promoter. (a) *Agrobacterium* vector for stable integration of the ZmMDHpro:Ems26+. The vector also contained a blue fluorescence gene (Cyan) as a visible marker regulated by the maize END2 promoter (END2). The disrupted DsRED gene (H2Bpro:RF‐FP) contained duplicated DsRED gene fragments (RF, FP) separated by a 136‐bp spacer containing the Ems26+ recognition site (position identified by the black bar above spacer sequence). (b) Blue and red fluorescence overlay image of rice calli containing stably integrated ZmMDHpro:Ems26+ after treatment at 37 °C for 48 h. Images captured 7 days after treatment. (c) Deletions in *OsMs26* achieved via targeted mutagenesis. Shown in the reference sequence, the 22‐nt Ems26+ recognition sequence is highlighted in grey and yellow. The latter indicates the 4 bp overhang, 5′‐GTAC‐3′, produced by nuclease cutting and is highlighted throughout for orientation purposes. Nucleotides shown are deleted sequence in seven of eight identified mutations, one larger deletion not shown. ZmMDH, Maize Mannitol dehydrogenase promoter; Ems26+, homing endonuclease targeting Ms26 target site; E, CaMV 35S promoter; H2B, maize Histone 2B promoter.

Given the ability to successfully direct mutations to the rice *Ms26* ortholog that confer a male sterile phenotype, the requirement of *Ms26* for pollen development in sorghum and wheat was investigated. Relative to maize and rice, transformation of sorghum and wheat is challenging (Casas *et al*., 1997; Zhao *et al*., 2000; Zhu *et al*., 1998). Moreover, gene editing in wheat is complicated due to the size and polyploid nature of the genome. Thus, as an alternative to biolistic delivery of Ems26+, an *in planta* method to stimulate targeted mutagenesis in sorghum and wheat was devised. This strategy depended on the generation of plants containing a stably integrated, temperature‐regulated Ems26+ expression cassette delivered on a T‐DNA vector by *Agrobacterium*‐mediated transformation. Ems26+ was placed under the transcriptional control of a maize temperature‐regulated promoter, MDH (ZmMDHpro:Ems26+) (Svitashev *et al*., 2015), which allowed for temperature‐inducible expression of the nuclease in plant tissues. In this strategy, immature embryos from plants containing conditionally expressed Ems26+ would be heated at 37 °C for 24 h to generate mutations. This T‐DNA also contained a blue fluorescence gene (Cyan) regulated by an embryo‐preferred END2 promoter (ZmEnd2pro:Cyan). This reporter served as a visible marker for selection of transformed callus, fertilized immature embryos and mature seed. A disrupted copy of the DsRED gene transcriptionally regulated by a maize Histone 2B promoter was also present in this T‐DNA to monitor endonuclease activity. This nonfunctional colour marker consisted of duplicated DsRED gene fragments (RF‐FP) with a 369‐bp overlap separated by a 136‐bp spacer containing the Ems26+ recognition site (Text S2). DSBs in RF‐FP would either be repaired by NHEJ or intramolecular recombination within the repeated RF‐FP fragments (Figure 4a). Duplicated gene coding regions interrupted by a DSB target site(s) have been demonstrated to be useful visual reporters to detect nuclease activity (Puchta and Hohn, 2012). As such, one outcome of a DSB at the target site would be to stimulate intramolecular recombination within the repeated RF‐FP restoring DsRED gene function as revealed by the appearance of red‐fluorescing callus cells against a background of blue fluorescence. Thus in contrast to control callus where no red fluorescence would be expected, the appearance of red‐fluorescing cells in treated samples would demonstrate activity of Ems26+ endonuclease and suggest potential cutting and, therefore, mutagenesis within the endogenous *Ms26* gene.

To test this scheme, rice plants were generated to contain a stable, randomly integrated ZmMDHpro:Ems26+ expression cassette as described above in the rice genome. Four independently transformed plants were allowed to self‐pollinate, and blue‐fluorescing seed (ZmEnd2pro:Cyan) were selected to initiate rice calli (Hervé and Kayano, 2006; Toki, 1997). Calli from these four events were allowed to grow at 26 °C (control) for 30 days, whereupon a portion of callus from each event was then incubated at 37 °C (treated) for 48 h. The temperature‐treated calli were returned to 26 °C, upon which control and treated samples were examined for the appearance of red‐fluorescing sectors. Over the course of 2 weeks, red sectors were readily visible in three of the four independent callus isolates treated at 37 °C (Figure 4b) and none in the control calli. Detection of red fluorescence indicated DSBs as a result of active Ems26+; therefore, genomic DNA was isolated from several red‐sectoring callus events and screened for mutations at the endogenous *OsMs26* gene. A DNA fragment spanning a portion of the *OsMs26* gene was amplified from control and temperature‐treated calli and screened using the *Bsi*WI digestion assay. Analysis of amplified products from the three callus events demonstrated RF‐FP repair and revealed *Bsi*WI‐resistant fragments present only in genomic DNA derived from these callus sectors incubated at 37 °C. Uncut amplified PCR products from control and temperature‐treated callus DNA were sequenced. Analysis of PCR products amplified from callus sectors maintained at 26 °C revealed only wild‐type *OsMs26* sequences. In contrast, only four of the 16 PCR products derived from callus exposed to 37 °C contained wild‐type *OsMs26* sequences. The remaining 12 products revealed mutations across the Ems26+ recognition site, which consisted of several independent deletions (Figure 4c). These observations demonstrated that a stably integrated and conditionally regulated endonuclease could be used in rice to generate site‐specific mutations and, thus, it has the potential to perform similarly in other monocots such as sorghum and wheat.

### Mutations in the sorghum *Ms26* ortholog confer male sterility

To determine whether the ortholog of maize *Ms26* in *S. bicolor* was required for pollen formation, Ems26+ was used to direct mutations to the conserved recognition site in the 5th exon of this single‐copy gene (*SbMs26*) present on Chromosome 1 (Sb01g045960). Immature embryos from *S. bicolor* [Tx430 (Miller, 1984)] were transformed with the ZmMDHpro:Ems26+ T‐DNA using *Agrobacterium*‐mediated delivery. Six independent blue‐fluorescing callus sectors, indicative of the presence of a stably integrated copy of ZmMDHpro:Ems26+ T‐DNA, were used to generate sorghum plants (approximately 2–12 plants per callus sector). Intactness and copy number of the T‐DNA insertion were determined in these plants by DNA hybridization analysis (Southern, 1975) while amplification and sequencing of genomic DNA confirmed the presence of only wild‐type *SbMs26* alleles in these plants. In addition, no red fluorescence was detected when progeny seed were screened visually. The presence of only wild‐type *SbMs26* alleles and the absence of red‐fluorescing seed suggested that the Ems26+ was not activated in these T0 plants. Several plants containing single‐copy ZmMDHpro:Ems26+ T‐DNA insertions were allowed to self‐pollinate, and blue‐fluorescing immature embryos (10 plants, 16 embryos per plant, representing four independent T‐DNA insertions) were harvested 10–15 days postfertilization to be used for *SbMs26* mutational analysis. Visual inspection of these harvested embryos revealed that there were no red‐fluorescing sectors or red‐fluorescing embryos present. These blue‐only‐fluorescing embryos were incubated at 26 °C or 37 °C for 48 h and examined for RF‐FP repair. In contrast to control embryos maintained at 26 °C, red‐fluorescing sectors were readily detected to varying degrees in all embryos incubated at 37 °C indicating the presence of active Ems26+. Calli from embryos treated at 37 °C were grown for 4 weeks and used to regenerate plants that were subsequently screened for mutations at the *SbMs26* target site on Chromosome 1. In total, approximately 20% of the plants (*n* = 440) regenerated from these four independent T‐DNA insertion events revealed heterozygous mutations within the *SbMs26* gene when screened by PCR amplification and digestion with *Bsi*WI. Although expected at a low frequency, biallelic mutations were not detected in these sorghum experiments. Similar to the experiments in rice, DNA sequence analysis of these PCR products (*n* = 50 independent *Bsi*WI‐resistant PCR fragments) revealed a variety of mutations across the *SbMs26* region that consisted of deletions and insertions. In total, 16 nonidentical mutations were identified in these regenerated sorghum plants (Figure S3 and Text S3).

**Figure 5:**
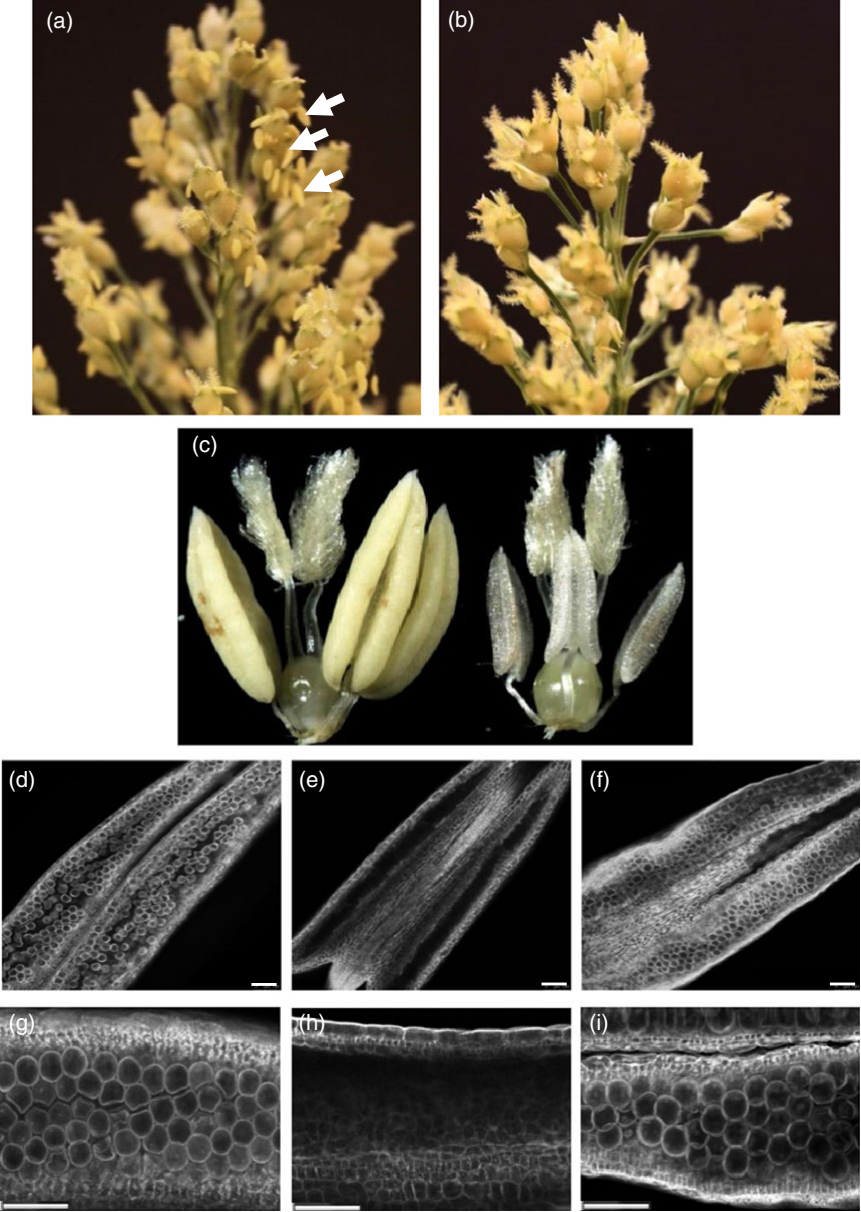
Male sterile sorghum generated from targeted mutagenesis of *SbMs26* gene. (a) Panicles from male fertile sorghum plants (*SbMs26/Sbms26*‐Δ78) with extruded anthers (white arrows). (b) Panicles from male sterile sorghum plants (*Sbms26*‐Δ78/*Sbms2*‐Δ78). (c) Floret from male fertile (left) and from male sterile (*Sbms26*/*Sbms26*) sorghum plants (right). (d‐i) Images of sorghum anthers derived from wild‐type plants (d and g), homozygous *Sbms26*/*Sbms26* plants (e and h), and *Sbms2*6/*Sbms26* plants containing ZmMs26 complementation vector (f and i). Magnification bar = 100 μM.

Sorghum plants containing heterozygous mutations at *SbMs26* were allowed to flower, and seed from self‐pollinated plants was advanced to examine male fertility phenotype in progeny plants. T1 progeny containing heterozygous *SbMs26* mutations [36‐bp deletion (*Sbms26*‐Δ36), 59‐bp deletion with 11‐bp insertion (*Sbms26*‐Δ59 + I11) and 78‐bp deletion (*Sbms26*‐Δ78)] were phenotypically identical to wild‐type sorghum Tx430 plants and were male fertile (Table 2). As shown in Figure 5a, panicles from *SbMs26*/*Sbms26*‐Δ78 plants extruded anthers that contained pollen and had begun to set seed as demonstrated by the appearance of collapsed stigmas after anthesis. Homozygous *Sbms26* plants were also normal in stature and development with the exception of being completely male sterile: *Sbms26*‐Δ78 plants extruded small shrivelled anthers, did not shed pollen and did not set seed upon self‐pollination (Figure 5b and c). Upon closer examination, pollen was easily detected in anthers from *SbMs26*/*Sbms26*‐Δ78 plants (Figure 5d and g), while pollen was not observed in anthers from *Sbms26*‐Δ78/*Sbms26*‐Δ78 plants (Figure 5e and h). In summary, all *SbMs26*/*Sbms26* plants were male fertile, while all *Sbms26*/*Sbms26* plants were male sterile (Table 2). These male sterile plants were female fertile as demonstrated by their ability to set seed when pollinated with wild‐type sorghum pollen.

**Table 2:** Male fertility phenotypes associated with sorghum mutants and complementation of male sterility using maize *Ms26* gene

		*SbMs26*/*Sbms26*		*Sbms26*/*Sbms26*	
Mutation	Plants	Male fertile	Male sterile	Male fertile	Male sterile
*Sbms26*‐Δ36	6	3	0	0	3
*Sbms26*‐Δ78	6	3	0	0	3
*Sbms26*‐Δ59 + I11	6	3	0	0	3

Complementation data					
Mutation	Plants	*Sbms26*/*Sbms26*		*Sbms26*/*Sbms26 *+ *ZmMs26*	
		Male fertile	Male sterile	Male fertile	Male sterile
*Sbms26*‐Δ36	10^a^	0	4	6	0
*Sbms26*‐Δ78	15^b^	0	10	5	0

*SbMs26*, sorghum *Ms26* ortholog; *ZmMs26*, maize *Ms26* gene.

a Includes events #3 and #4.

b Includes events #1, #2, and #3.

To further investigate that the male sterile phenotype observed in these sorghum mutant plants was directly due to deletions at *SbMs26*, restoration of fertility was tested using a copy of the maize *Ms26* gene (also referred to as *ZmMs26*) introduced by transformation. Wild‐type sorghum plants were generated to contain a single copy of the *ZmMs26* gene introduced on a T‐DNA expression cassette and crossed with plants carrying either the *Sbms26*‐Δ36 or the *Sbms26*‐Δ78 deletion mutation. From these crosses, F1 plants were screened and those that contained both the *Sbms26* mutation and *ZmMs26* were allowed to self‐pollinate to generate F2 seeds. F2 plants were grown under greenhouse conditions and genotyped for the *Sbms26* mutation and presence of *ZmMs26*. A set of F2 plants homozygous for the *Sbms26* mutation but segregated for the *ZmMs26* expression cassette were selected. Plants homozygous for *Sbms26*‐Δ36 or *Sbms26‐*Δ78 but lacking *ZmMs26* were male sterile; anthers did not contain pollen, and plants did not set seed upon self‐pollination (Table 2 and Table S1). However, when *ZmMs26* was present, these homozygous *Sbms26* mutant plants were male fertile (Table 2) with pollen development comparable to wild type (Figure 5f and i). The requirement of Sb01g045960 for pollen development in sorghum suggests that this gene is the ortholog of the maize male fertility gene *Ms26*.

### Ems26+‐targeted mutations in the wheat *Ms26* ortholog

As described above, the maize Ms26 polypeptide sequence was used to search the translated hexaploid wheat genome database (Chinese spring) (Consortium, 2014) for homologous sequences. A 4.2‐kb contig derived from Chromosome 4AS was found capable of encoding a 550 amino acid polypeptide having 91% sequence similarity to maize Ms26 protein (Figure S1 and Text S4). Additional BLAST (NCBI, Bethesda, MD, USA) search yielded wheat 4BL and 4DL contigs that only partially overlapped the region corresponding to the 2nd (4DL) and last exons (4BL and 4DL) of the 4AS sequence. To ascertain whether the Ems26+ recognition site was conserved across all three wheat genomes, UNIMS26 primers were used to amplify genomic DNA from the hexaploid spring wheat cultivar Fielder. DNA sequence analysis of these amplified products, when compared to the contigs described above, suggested that the 22‐nt Ems26+ recognition site was conserved. The A‐, B‐ and D‐genomic copies of the wheat *Ms26* ortholog (referred to as *TaMs26*) were assigned to the Fielder genome (Figure S4). Distinguishing features within this amplified region include a six‐nucleotide, two‐amino acid gap; functional relevance of this sequence difference is not yet known, but interestingly is found in a region that is diverse across maize, sorghum, rice and wheat Ms26 protein alignments (Figure S5).

**Figure 6:**
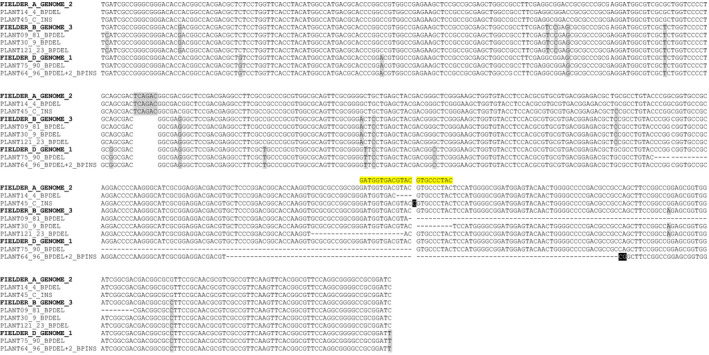
*TaMs26* gene mutations isolated from Fielder wheat A‐, B‐ and D‐genomes via targeted mutagenesis. Ems26+ recognition sequence is highlighted in yellow above the A‐genome sequence. Deleted nucleotide sequence is represented by a hyphen (‘‐’). Inserted nucleotide sequence is in white font and highlighted in black. Sequence differences between the A‐, B‐ and D‐genomes are highlighted in grey. White space indicates a gap in sequence.

To examine whether the Ems26+ endonuclease was competent to generate targeted mutations at the *TaMs26* gene orthologs in hexaploid wheat, Fielder plants containing the ZmMDHpro:Ems26+ T‐DNA were generated by *Agrobacterium*‐mediated transformation. Immature Fielder embryos were used as transformation targets. Blue‐fluorescing callus sectors indicative of T‐DNA integration were selected to initiate plant regeneration. Young wheat plantlets grown under greenhouse conditions were analysed for ZmMDHpro:Ems26+ T‐DNA copy number by qPCR. Four independently transformed wheat plants containing single‐ or low‐copy ZmMDHpro:Ems26+ T‐DNA were allowed to self‐pollinate and embryos were harvested 15 days postfertilization. Blue‐fluorescing embryos (*n* = 12) isolated from each plant were either held in the dark at 26 °C (control, *n* = 4) or incubated at 37 °C for 24 h (treated, *n* = 8), whereupon the treated embryos were returned to 26 °C and allowed to grow in the dark. As observed for the rice and sorghum experiments, approximately 72 h after treatment, wheat embryos incubated at 37 °C began to develop red‐fluorescing sectors, consistent with the presence of active Ems26+ endonuclease (Figure S6). No red‐fluorescing sectors were detected in control, nontreated wheat embryos. To ascertain whether Ems26+ generated mutations at endogenous *TaMs26* sequences, total genomic DNA was isolated from temperature‐treated and control embryos and amplified using UNIMS26 primers. Deep sequencing analysis of these amplicons (approximately 5 million reads per sample) revealed that 1.6% of the amplicon reads from the temperature‐treated embryos contained mutations as compared to <0.007% from control embryos (Table S2 and Figure S7). Callus from embryos treated at 37 °C and containing ZmMDHpro:Ems26+ was used to regenerate 121 plants that were then screened for mutations using the *Bsi*WI digestion assay; ten wheat plants contained *Bsi*WI digestion‐resistant amplicons. DNA sequencing of these resistant PCR products identified seven different mutations within the Ems26+ recognition site consisting of a single‐nucleotide addition and deletions of various sizes; a nonoverlapping subset of these mutations was found within each genome copy of *TaMs26* (Figure 6). Plants containing *TaMs26* mutations were allowed to self‐pollinate for progeny analysis. Inheritance analysis determined that, with the exception of the single‐C‐nucleotide‐insertion mutant (no seed set), mutations in all three genomes were sexually transmitted to progeny plants. These progeny plants containing single‐genome, homozygous mutations in the A‐, B‐ or D‐genomic copies of *TaMs26* were phenotypically normal and male fertile (Table S3). Together these experiments demonstrated that a conditionally expressed Ems26+ was capable of generating targeted mutations at the *TaMs26* gene *in planta* and that single‐genome mutants in this P450 are not sufficient to confer a male sterile phenotype in the spring wheat cultivar Fielder. Studies that genetically combine these different A‐, B‐ and D‐genome mutations are in progress to determine the requirement of *Ms26* for pollen development in wheat.

## Discussion

The ability to perform targeted mutagenesis in plants is a valuable tool for gene functional analysis and generation of genetic diversity. In this report, targeted mutagenesis of the monocot male fertility gene, *Ms26*, was accomplished in three different plant species using either a constitutively or conditionally expressed custom designed I‐*Cre*I endonuclease, Ems26+. In rice, approximately 7% of the primary plants contained mutations across the highly conserved Ems26+ target site; these mutations ranged from single‐nucleotide deletions to large insertions. Similar to mutations identified by classical mutagenesis of maize and rice, targeted mutations to the rice *Ms26* ortholog also resulted in complete male sterility by preventing the formation of pollen in homozygous recessive mutant plants. During these studies, it was determined that plants carrying a constitutively expressed Ems26+ endonuclease did not promote mutagenesis in subsequent generations of plants suggesting that mutations occurred early in the plant transformation process and functional endonucleases were not stably transmittable. In contrast, when Ems26+ was introduced into transformed plants and placed under the transcriptional control of a conditional promoter, the endonuclease was functional in progeny sorghum and wheat plants.

In previous reports, the low frequency of stable event recovery has been suggested to be an indicator of cytotoxicity due to off‐target recognition for ZFNs in human cell lines and plants (Cornu *et al*., 2007; Szczepek *et al*., 2007; Townsend *et al*., 2009). This suggestion is supported by the observation that in maize and rice experiments, in contrast to control experiments that did not introduce UBIpro:Ems26+, event recovery was lower when Ems26+ was delivered by biolistic transformation. Although it cannot be ruled out that reduced transformation frequency in these experiments was simply a consequence of the high concentration of vectors containing Ems26+ delivered by particle bombardment when compared to *Agrobacterium* transformation, differences in delivery and regulation of Ems26+ were important design considerations for these targeted mutagenesis experiments. The establishment of pre‐integrated and conditionally regulated endonuclease was one strategy that mitigated potential toxicity by taking advantage of a promoter capable of induction at higher temperatures and the temperature‐dependent biochemical activity of this class of endonucleases (Wang *et al*., 1997). In our studies, Ems26+ demonstrated increased nuclease activity at 37 °C both *in vitro* and *in planta*. Thus, short pulses at elevated temperatures during tissue culture or plant growth resulted in both increased transcription and nucleolytic activity of the Ems26+ gene and protein, respectively. This dual switch was an effective method for controlling DSB activity in these experiments and has been exploited as a means to rapidly generate large collections of allelic variants for gene discovery.

Pollen formation in plants is highly conserved at the biochemical and genetic level (for review see Shi *et al*., 2015). A number of pollen‐ and anther‐specific genes have been identified whose function is conserved across plants. The maize *Ms26* and rice *OsCYP704B2* genes encode a cytochrome P450 that plays a critical role in male fertility. In this study, conservation of sequence within these orthologous genes was used to introduce targeted mutations in rice, sorghum and wheat. Importantly, this is the first isolation and functional characterization of a male fertility gene in sorghum, a close relative to maize. As in rice, sorghum plants containing homozygous recessive mutations in the ortholog of the maize *Ms26* gene were male sterile. Moreover, a transformed copy of the maize *Ms26* gene was able to restore fertility in these sorghum mutant plants. The ability to restore fertility in these sorghum mutants using a maize *Ms26* gene further supports biochemical conservation of P450 function.

This observed functional conservation is particularly relevant to the foundational experiments described in this report where the orthologs of the maize *Ms26* gene were targeted in wheat. To date, there are only two recessive male fertility mutants that have been described in *Triticum*:* Ms1* and *Ms5* (Whitford *et al*., 2013). Given the high sequence similarity across maize, sorghum and rice, it is likely that orthologs of *Ms26* in wheat are also required for pollen development. In contrast to these diploid species, wheat is an allohexaploid. In this study, it was observed that single‐genome homozygous *TaMs26* mutations in hexaploid wheat did not confer a male sterility phenotype. These results suggest that no single *TaMs26* genomic copy from the A‐, B‐ or D‐genome is essential to fertility in wheat, as the other wild‐type *TaMs26* copies present in these plants are likely to function to maintain pollen development and a male fertility phenotype. It is interesting to speculate that despite the high amino acid sequence conservation across all three wheat genome copies, functional differences may be revealed and associated with relatively minor sequence changes, in particular within the regions of amino acid diversity (Figure S5). Thus, combining these *TaMs26* gene mutations via genetic crosses will allow examination of the effects of various combinations of homozygous *TaMs26* mutations in the three genomes of hexaploid wheat (*i.e*. in one, two or all three single genomes) on male fertility.

In this report, targeted mutagenesis of the monocot male fertility gene, *Ms26*, was made possible by advances of genomics, gene editing and plant transformation that overcome two barriers in crop improvement. First, having the ability to specifically target and modify fertility genes would extend SPT systems to crops having perfect flowers enabling yield gains by heterosis not yet fully realized. Second, gene editing, when combined with advanced plant transformation methods, would allow for precision breeding of elite germplasm reducing the time needed for introgression of induced sequence diversity by backcrossing. Classical methods for introducing genetic diversity into plants include mutagenesis with chemical and physical agents or transposition. These nontargeted methods result in random and often additional and undesired changes in the genome. These approaches are both time‐consuming and resource‐intensive requiring multiple generations of backcrossing and selection to convert back to the original state of elite genetics for maximal crop yields. In contrast to these nontargeted approaches that slow discovery and crop improvement, adoption of the precision breeding innovations described above is essential to sustaining agriculture as population increases and as protection of natural resources becomes more challenging (Tian *et al*., 2016; Tilman *et al*., 2011; West *et al*., 2014). Targeted mutagenesis of the *Ms26* gene is a good example of how advanced technologies could be deployed to rapidly and precisely generate mutant fertility alleles in elite germplasm of maize, rice, sorghum and potentially wheat. While not exclusively considered a replacement for classical breeding methods, these advances could have a positive impact and change modern agriculture. As with classical mutagenesis methods, should undesired genome modifications occur (*i.e*. random integration of plasmid DNA or off‐target mutations), these changes could be easily bred away by genetic crosses to elite parental lines, which would eliminate unwanted genome alterations yet maintain targeted sequence variation. In addition, high‐resolution sequencing is particularly useful in the design and testing of nucleases with unique recognition and cutting specificity, thus further improving and advancing gene editing as an accelerated breeding tool.

## Experimental procedures

### DNA methods

Standard DNA techniques as described in Sambrook *et al*. (1989) were used for vector construction. The Ems26+ was maize codon‐optimized and designed to include an amino‐terminal nuclear localization signal SV40 (MAPKKKRKV, nucleotide position +1–30) and the potato ST‐LS1 intron [Accession number X04753 (nucleotides 2837–2892)] at nucleotide position +403–591 (Text S5). The chemically synthesized Ems26+, contained on a 1.280‐kb NcoI‐KpnI DNA fragment, was subcloned into PHP17720 (Sabelli *et al*., 2009) digested with NcoI‐KpnI that links Ems26+ to a maize constitutive ubiquitin‐1 promoter, including the first intron [−899 to +1092 (Christensen *et al*., 1992)], while transcription is terminated by the addition of the 3′ sequences from the potato proteinase inhibitor II gene (PinII) [nucleotides 2–310 (An *et al*., 1989)] to generate UBIpro:Ems26+ vector. To generate ZmENDpro:PAT RFP fusion plasmid, the maize END2 promoter [Endosperm‐ and embryo‐expressed promoter from maize; Chromosome 8, nt 171120325‐171119383 Maize (B73) Public Genome Assembly (AGP_v3.8)] was fused to the MoPAT‐DsRED (a translational fusion of the bialophos resistance gene, phosphinothricin‐*N*‐acetyl‐transferase, and the red fluorescent protein DsRED) expression cassette previously described in Ananiev *et al*. (2009) with transcription terminated by the PinII terminator described above. To enhance expression of this expression cassette, a 485‐bp fragment of the CaMV 35S promoter was inserted upstream of the ZmEnd2 promoter as described in Unger *et al*. (2001).

To generate a conditionally expressed Ems26+, a 1048‐bp fragment of temperature‐regulated promoter from the maize mannitol dehydrogenase gene [MDH; temperature‐inducible maize promoter; Chromosome 2, nt 8783228–8784275 Maize (B73) Public Genome Assembly (AGP_v3.2)] was modified to contain an *Rca*I restriction site at the 3′ end of the sequence (Svitashev *et al*., 2015) to accommodate translational fusion to the maize‐optimized Ems26+, producing the ZmMDHpro:Ems26+ expression cassette. To construct the stably integrated Ems26+ expression cassette, a T‐DNA vector containing ZmMDHpro:Ems26+ was generated and was identical to that described in Svitashev *et al*. (2015) with the exception that the ZmMDHpro:Ems26+ present on a 3.339‐kb *Hin*dIII‐*Pac*I fragment replaced the UBIpro:Cas9 portion of this vector. In addition, the duplicated DsRED fragments in this gene were separated by a 136‐bp spacer that contained sequences for recognition by the Ems26+ endonuclease (Text S2). This plasmid was introduced into *Agrobacterium* strain LBA4404 (Komari *et al*., 1996) by electroporation using a Gene Pulser II (Bio‐Rad Laboratories, Inc., Hercules, CA, USA) as described by Gao *et al*. (2010) to generate *Agrobacterium* strains for plant transformation.

To generate the *ms26* fertility complementation T‐DNA vector, the *Ms45* gene in PHP24490 (Wu *et al*., 2015) was replaced by a 3.9‐kb DNA fragment [Chromosome 1, nt 4509689–14506399 Maize (B73) RefGen_v2 (MGSC)], which contained the wild‐type *Ms26* gene. The LTP2 promoter present in this T‐DNA was also replaced by the maize END2 promoter as described above to transcriptionally regulate the RFP gene in this plasmid. The final T‐DNA cassette, pPG47::Bt1:ZmAA1//Ms26//35SEN‐pEND2::DsRed2, was introduced into *Agrobacterium* strain LBA4404 as described above.

For PCR and DNA sequence analysis, DNA was extracted from a small amount (0.5 cm in diameter) of callus tissue or two leaf punches (four leaf punches for wheat) as described in Gao *et al*. (2010). PCR was performed using REDExtract‐N‐Amp^™^ PCR ReadyMix (Sigma‐Aldrich Co., St. Louis, MO, USA) or Phusion^®^ High‐Fidelity PCR Master Mix (New England Biolabs Inc., Ipswich, MA, USA) according to the manufacturer's recommendations. Ms26 target site mutations were identified by amplification of the region by PCR using the primer pair UNIMS26 5′‐2 (5′‐GACGTGGTGCTCAACTTCGTGAT‐3′) and UNIMS26 3′‐1 (5′‐GCCATGGAGAGGATGGTCATCAT‐3′) and digestion of the amplified products with *Bsi*WI, the DNA restriction enzyme that recognizes the sequence 5′‐CGTACG‐3′, as described in Djukanovic *et al*. (2013). Products of these reactions were electrophoresed on 1% agarose gels and screened for *Bsi*WI digestion‐resistant bands indicative of mutations at the Ms26 target site. PCR‐amplified fragments were also subcloned for DNA sequence analysis using the pCR2.1‐TOPO cloning vector (Thermo Fisher Scientific, Waltham, MA, USA).

### Deep sequencing

Total genomic DNA was extracted from the temperature‐treated and nontreated calli transformed with the Ems26+ homing endonuclease using a modified CTAB method (Unger *et al*., 2001). The region surrounding the Ms26 target site was PCR‐amplified with Phusion^®^ High‐Fidelity PCR Master Mix (New England Biolabs) adding on the sequences necessary for amplicon‐specific barcodes and Illumina sequencing using ‘tailed’ primers through two rounds of PCR. The primers used in the primary PCR Ms26 reaction were Ms26F (5′‐CTACACTCTTTCCCTACACGACGCTCTTCCGATCTAACCCGCGGAGGACGACGTGCTC‐3′) and Ms26R (5′‐CAAGCAGAAGACGGCATACGAGCTCTTCCGATCTCGTCGGGGCCCCAGTTGTAC‐3′), while the primers used in the secondary PCR reaction were F2 (5′‐AATGATACGGCGACCACCGAGATCTACACTCTTTCCCTACACG‐3′) and R2 (5′‐CAAGCAGAAGACGGCATA‐3′). Genomic DNA extracted from leaves of untransformed Fielder plants served as a negative control. The resulting PCR amplifications were concentrated using a Qiagen MinElute^®^ PCR purification spin column (QIAGEN, Inc., Venlo, Netherlands), electrophoresed on a 2% agarose gel, and amplifications were then excised and purified with a Qiagen gel extraction spin column. The concentration of the gel‐purified amplifications was measured with a Hoechst dye‐based fluorometric assay, combined in an equimolar ratio, and single‐read 100‐nt‐length deep sequencing was performed on an Illumina Genome Analyzer IIx (ELIM Biopharmaceuticals, Inc., Hayward, CA, USA) with a 30%–40% (v/v) spike of PhiX control v3 (Illumina, Inc., San Diego, CA, USA) to off‐set sequence bias. Only those reads with a ≥1 nucleotide indel arising within a 6‐nt window centred over the expected site of cleavage and not found in a similar level in the negative control were classified as NHEJ mutations. The total numbers of NHEJ mutations were then used to calculate the % mutant reads based on the total number of reads of an appropriate length containing a perfect match to the barcode and forward primer.

### Plant methods

Rice, sorghum and wheat plant transformation and tissue culture details are outlined in Data S1**.**

### Microscopy

Sorghum samples were dissected to remove the glumes and expose the anthers, and fixed in 2% paraformaldehyde and 4% glutaraldehyde overnight under vacuum (10 psi). After fixation, the samples were rinsed three times in 1× PBS (Phosphate Buffer Solution). Samples were then cleared in graded TDE (2, 2′‐Thiodiethanol) series (10%, 25%, 50% and 97%), and the 97% was repeated to assure optimal clearing. Samples were mounted in 97% TDE and the coverslip was sealed with nail polish. Confocal images were taken with the Leica (Wetzlar, Germany) TCS SPE using the solid‐state 405 nm with the DAPI setting (emission: 430–480); the 488 nm with the GFP setting (emission: 500–600); and the 561 nm with the DRED setting (emission: 650–700) laser lines and merged using the Leica LAS X software. Final image adjustments were accomplished with Adobe Systems Photoshop CS6 (San Jose, CA).

## Conflict of interest

AMC and JY are inventors on pending applications on this work and are current employees of DuPont Pioneer who owns the pending patent applications. MS, GB, LF, MK, M‐JC, NS and SS are current or former employees of DuPont Pioneer.

## Supporting information


**Figure S1** Ms26 protein sequence is highly conserved across crop species.
**Figure S2** *Bs*iWI restriction enzyme resistance assay screen for *OsMs26* mutations.
**Figure S3** *SbMs26* gene mutations in sorghum achieved via targeted mutagenesis.
**Figure S4** DNA sequences corresponding to the IWGSC contigs containing the wheat 4AS, 4BL and 4DL genomic regions orthologous to maize *Ms26* gene and aligned with DNA sequences from Fielder.
**Figure S5** Predicted protein translation products of amplicons derived from UNIMS26 primers.
**Figure S6** Harvested Fielder wheat embryo containing Ems26+ under transcriptional control of a maize temperature‐regulated promoter.
**Figure S7** Top 10 amplicon sequences containing mutations from genomic DNA derived from Fielder wheat callus transformed with ZmMDHpro:Ems26+.
**Table S1** Restoration of male fertility to homozygous *Sbms26*/*Sbms26* plants with a transformed copy of maize *ZmMs26* was achieved using four nonidentical events in two different *Sbms26* mutant backgrounds.
**Table S2** Percent (%) mutant reads at the wheat Ems26+ target locus identified by deep sequencing.
**Table S3** Male fertility phenotype for wheat *TaMs26* mutant lines.
**Text S1** 4.194‐kb region containing wheat *Ms26* ortholog IWGSC 4AS.
**Text S2** Spacer sequence (136 bp) containing multiple nuclease target sites.
**Text S3** Nucleotide sequence of Ems26+ target site (underlined) present in Chromosome 7 in *Sorghum bicolor* Tx430.
**Text S4** Predicted 550 amino acid sequence encoded by wheat *Ms26* ortholog IWGSC 4AS coding region.
**Text S5** Maize‐optimized Ems26+ coding region.
**Data S1** Experimental procedures detailing plant transformation and tissue culture methods.
