# Supplementary material for: Targeted mutagenesis of a conserved anther‐expressed P450 gene confers male sterility in monocots
Source: Plant Biotechnol J. 2016 Oct 14;15(3):379–89. doi: 10.1111/pbi.12633 (PMC5316918; doi:10.1111/pbi.12633)
Supplement: Supplementary file 1 — Figure S1 Ms26 protein sequence is highly conserved across crop species. Figure S2 BsiWI restriction enzyme resistance assay screen for OsMs26 mutations. Figure S3 SbMs26 gene mutations in sorghum achieved via targeted mutagenesis. Figure S4 DNA sequences corresponding to the IWGSC contigs containing the wheat 4AS, 4BL and 4DL genomic regions orthologous to maize Ms26 gene and aligned with DNA sequences from Fielder. Figure S5 Predicted protein translation products of amplicons derived from UNIMS26 primers. Figure S6 Harvested Fielder wheat embryo containing Ems26+ under transcriptional control of a maize temperature‐regulated promoter. Figure S7 Top 10 amplicon sequences containing mutations from genomic DNA derived from Fielder wheat callus transformed with ZmMDHpro:Ems26+. Table S1 Restoration of male fertility to homozygous Sbms26/Sbms26 plants with a transformed copy of maize ZmMs26 was achieved using four nonidentical events in two different Sbms26 mutant backgrounds. Table S2 Percent (%) mutant reads at the wheat Ems26+ target locus identified by deep sequencing. Table S3 Male fertility phenotype for wheat TaMs26 mutant lines. Text S1 4.194‐kb region containing wheat Ms26 ortholog IWGSC 4AS. Text S2 Spacer sequence (136 bp) containing multiple nuclease target sites. Text S3 Nucleotide sequence of Ems26+ target site (underlined) present in Chromosome 7 in Sorghum bicolor Tx430. Text S4 Predicted 550 amino acid sequence encoded by wheat Ms26 ortholog IWGSC 4AS coding region. Text S5 Maize‐optimized Ems26+ coding region. Data S1 Experimental procedures detailing plant transformation and tissue culture methods. [file PBI-15-379-s001.docx]

Targeted Mutagenesis of a Conserved Anther-Expressed P450 Gene Confers Male Sterility in Monocots

**Supporting Information**

**Table S1** Restoration of male fertility to homozygous *Sbms26*/*Sbms26* plants with a transformed copy of maize *ZmMs26* was achieved using four non-identical events in two different *Sbms26* mutant backgrounds.

| **Event** | ***ZmMs26*** | ***Sbms26-*Δ36** | | ***Sbms26-*Δ78** | |
| --- | --- | --- | --- | --- | --- |
|  |  | **Sterile (n)** | **Fertile (n)** | **Sterile (n)** | **Fertile (n)** |
| #1 | + |  |  | 0 | 2 |
| #1 | - |  |  | 3 | 0 |
| #2 | + |  |  | 0 | 1 |
| #2 | - |  |  | 4 | 0 |
| #3 | + | 0 | 3 | 0 | 2 |
| #3 | - | 1 | 0 | 3 | 0 |
| #4 | + | 0 | 3 |  |  |
| #4 | - | 3 | 0 |  |  |

**Table S2** Percent (%) mutant reads at the wheat Ems26+ target locus identified by deep sequencing.

| **System** | **Heat treatment** | **Total mutant reads (n)** | **Total reads (n)** | **Mutant reads (%)** |
| --- | --- | --- | --- | --- |
| Untransformed wheat genomic DNA control | N/A | 19 | 3,989,749 | 0.00% |
| Wheat callus transformed with Ems26+ | None | 304 | 4,069,593 | 0.007% |
| Wheat callus transformed with Ems26+ | 37ᵒC for 24 h | 64,158 | 4,055,925 | 1.58% |

**Table S3** Male fertility phenotype for wheat *TaMs26* mutant lines

| **Mutation** | **Sequence change** | **Genome** | ***TaMs26* allele** | **Male fertility phenotype** |
| --- | --- | --- | --- | --- |
| **1** | 4 bp deletion | A | *TaMs26^aΔ4/B/D^* | fertile |
| **2** | 9 bp deletion | B | *TaMs26^A/bΔ9/D^* | fertile |
| **3** | 81 bp deletion | B | *TaMs26^A/bΔ81/D^* | fertile |
| **4** | 23 bp deletion | B | *TaMs26^A/bΔ23/D^* | fertile |
| **5** | 90 bp deletion | D | *TaMs26^A/B/dΔ90^* | fertile |
| **6** | 96 bp deletion + 2 bp insertion | D | *TaMs26^A/B/dΔ96+2I^* | fertile |

Maize MEEAHLTP-ATPSPFFPLAGPHKYIALLLVVLSWILVQRWSLRKQKGPRSWPVIGATVEQLRNYHRMHDWLVGYLSRHRTVTVDMPFTSYTYIADPVNVEHVLKTNFTNYPKGIVYRSYMDVLLGDGIFNADGELWRKQRKTASFEFASKNLRDFSAIVFREYSLKLSGILSQASKAGKVVDMQELYMRMTLDSICKVGF

Sorghum MEEAHLMP ATP--LFPLAGLHKYIAILLVVLSWALVHRWSLRKQKGPRSWPVIGATLEQLRNYHRMHDWLVGYLSRHKTVTVDMPFTSYTYIADPVNVEHVLKTNFTNYPKGDVYRSYMDVLLGDGIFNADGELWRKQRKTASFEFASKNLRDFSANVFREYSLKLSGILSQASKAGKVVDMQELYMRMTLDSICKVGF

Rice MEEAHAMP VTSFFPVAGIHKLIAIFLVVLSWILVHKWSLRNQKGPRSWPIIGATVEQLKNYHRMHDWLVEYLSKDRTVTVDMPFTSYTYIADPVNVEHVLKTNFTNYPKGEVYRSYMDVLLGDGIFNADGEMWRKQRKTASFEFASKNLRDFSTVVFREYSLKLSSILSQACKAGRVVDMQELFMRMTLDSICKVGF

Wheat 4AS MEEAHHGMPSTTTAFFPLAGLHKFMAIFLVFLSWILVHWWSLRKQKGPRSWPVIGATLEQLRNYYRMHDWLVEYLSKHRTVTVDMPFTSYTYIADPVNVEHVLKTNFNNYPKGEVYRSYMDVLLGDGIFNADGELWRKQRKTASFEFASKNLRDFSTIVFREYSLKLRSILSQACKAGKVVDMQELYMRMTLDSICKVGF

Maize GVEIGTLSPDLPENSFAQAFDAANIIITLRFIDPLWRIKRFFHVGSEALLAQSIKLVDEFTYSVIRRRKAEIVEVRASGKQEKMKHDILSRFIELGEAGDDGGG--FGDDKSLRDVVLNFVIAGRDTTATTLSWFTHMAMSHPDVAEKLRRELCAFEAERAREEGVTLVLCGGADAD--DKAFAARVAQFAGLLTYDSLG

Sorghum GVEIGTLSPDLPENSFAQAFDAANIIVTLRFIDPLWRVKRFFHVGSEALLAQSIKLVDEFTYSVIRRRKAEIVEARASGKQEKMKHDILSRFIELGEAGDDGG---FGDDKSLRDVVLNFVIAGRDTTATTLSWFTHMAMSHPDVAEKLRRELCAFEAERAREEGVAVPCCGPDD----DKAFAARVAQFAGLLTYDSLG

Rice GVEIGTLSPDLPENSFAQAFDAANIIVTLRFIDPLWRLKKFLHVGSEALLEQSMKLVDDFTYSVIRRRKAEILQARASGKQEKIKHDILSRFIELGEAGGDEGGGSFGDDKSLRDVVLNFVIAGRDTTATTLSWFTYMAMTHPAVADKLRRELAAFEDERAREEGVALADAAGEA------SFAARVAQFASLLSYDAVG

Wheat 4AS GVEIGTLSPELPENSFAQAFDAANIIVTLRFIDPLWRVKKFLHVGSEALLEQSIKLVDEFTYSVIRRRKAEIVQARASGKQEKIKHDILSRFIELGEAGGDDGGSLFGDDKGLRDVVLNFVIAGRDTTATTLSWFTYMAMTHPAVAEKLRRELAAFEADRAREDGVALVPCSDSDGDGSDEAFAARVAQFAGLLSYDGLG

Maize KLVYLHACVTETLRLYPAVPQDPKGILEDDVLPDGTKVRAGGMVTYVPYSMGRMEYNWGPDAASFRPERWI-NEDGAFRNASPFKFTAFQAGPRICLGKDSAYLQMKMALAILFRFYSFRLLEGHPVQYRMMTILSMAHGLKVRVSRAV---

Sorghum KLVYLHACVTETLRLYPAVPQDPKGILEDDVLPDGTKVRAGGMVTYVPYSMGRMEYNWGPDAASFRPERWI-NEEGAFRNASPFKFTAFQAGPRICLGKDSAYLQMKMALAILFRFYSFQLLEGHPVQYRMMTILSMAHGLKVRVSRAV---

Rice KLVYLHACVTETLRLYPAVPQDPKGIVEDDVLPDGTKVRAGGMVTYVPYSMGRMEYNWGPDAASFRPERWLSGDGGAFRNASPFKFTAFQAGPRICLGKDSAYLQMKMALAILFRFYTFDLVEDHPVKYRMMTILSMAHGLKVRVSTSV---

Wheat 4AS KLVYLHACVTETLRLYPAVPQDPKGIAEDDVLPDGTKVRAGGMVTYVPYSMGRMEYNWGPDAASFRPERWI-GDDGAFRNASPFKFTAFQAGPRICLGKDSAYLQMKMALAILCRFFRFELVEGHPVKYRMMTILSMAHGLKVRVSRAPLA-

**Figure S1** Ms26 protein sequence is highly conserved across crop species. Sequence differences in rice (XP_015629295.1), sorghum (EER92794), and wheat 4AS (derived from Text S1) as compared to maize Ms26 (AAK52956.1) protein indicated as underlined amino acids. White space indicates a gap in sequence.

**1 2 3 4 5 6**


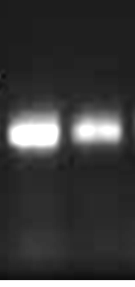


**U B U B U B**

**Figure S2** *Bsi*WI restriction enzyme resistance assay screen for *OsMs26* mutations. PCR amplification products from genomic DNA isolated from rice tissue containing wild-type (*OsMs26*/*OsMs26*; lanes 1, 2), heterozygous (*OsMs26*/*Osms26*; lanes 3, 4), and homozygous mutant (*Osms26*/*Osms26*; lanes 5, 6) *OsMs26* alleles. The panel is a composite of two photographs. Products of all lanes were electrophoresed on the same gel, with lane 5 and 6 products present at the far end of the gel and representative of products from homozygous mutant reactions as shown previously ([Djukanovic *et al.*, 2013](#_ENREF_4)). Undigested PCR amplicons (U) or amplicons resistant to digestion migrated as a single 653 bp DNA fragment; amplicons containing an intact sequence across the Ems26+ target site and digested with *Bsi*WI (B) yielded 376 bp and 277 bp DNA fragments when electrophoresed on 1% agarose gels.

**Figure S3** *SbMs26* gene mutations in sorghum achieved via targeted mutagenesis. Shown in the WT reference sequence, the 22 nt Ems26+ recognition sequence is highlighted in green and yellow. The latter indicates the four base-pair overhang, 5’-GTAC-3’, produced by nuclease cutting and is highlighted throughout for orientation purposes. For each mutant, deleted nucleotides are shown and inserted nucleotides are shown as underlined. Amino acid sequence corresponding to the *SbMs26* gene is provided as single letters above the WT nucleotide reference sequence. A second 22 nt Ems26+ target site was identified on Chromosome 7 in Tx430 sorghum variety (Text S3). Mutations at this second site on Sb Chromosome 7 were recovered at approximately the same frequency as at the Sb Chromosome 1 site. Plants containing homozygous mutations in SbChrom7 site were tested for plant stature and fertility phenotypes; these plants were phenotypically normal and both male and female fertile.

**Figure S4** DNA sequences corresponding to the IWGSC contigs containing the wheat 4AS, 4BL, and 4DL genomic regions orthologous to maize *Ms26* gene and aligned with DNA sequences from Fielder. 4BL and 4DL sequences are truncated within and 3’ proximal to the Ems26+ target site (yellow highlighted sequence), respectively. The sequence of wheat 4AS region is shown on the top line for reference; sequence identities are displayed as hyphens (‘-‘) and nucleotide polymorphisms are given. White space indicates a gape in sequence. The three non-sequence identical amplicon products (668 nt) from Fielder genomic DNA using UNIMS26 primer pairs are shown below their corresponding sequence identical wheat genomic contig.

Maize DVVLNFVIAGRDTTATTLSWFTHMAMSHPDVAEKLRRELCAFEAERAREEGVTLVLCGGAD--ADDKAFAARVAQFAGLLTYDSLGK

Sorghum DVVLNFVIAGRDTTATTLSWFTHMAMSHPDVAEKLRRELCAFEAERAREEGVAVPCCGPD----DDKAFAARVAQFAGLLTYDSLGK

Rice DVVLNFVIAGRDTTATTLSWFTYMAMTHPAVADKLRRELAAFEDERAREEGVALADAAGEA---S---FAARVAQFASLLSYDAVGK

FIELDER_A_GENOME_2 DVVLNFVIAGRDTTATTLSWFTYMAMTHPAVAEKLRRELAAFEADRAREDGVALVPCSDSDGDGSDEAFAARVAQFAGLLSYDGLGK

FIELDER_B_GENOME_3 DVVLNFVIAGRDTTATTLSWFTYMAMTHPAVAEKLRRELAAFESERAREDGVALVPCSDGE--GSDEAFAARVAQFAGLLSYDGLGK

FIELDER_D_GENOME_1 DVVLNFVIAGRDTTATTLSWFTYMAMTHPDVAEKLRRELAAFEAERAREDGVALVPCGDGE--GSDEAFAARVAQFAGFLSYDGLGK

Maize LVYLHACVTETLRLYPAVPQDPKGILEDDVLPDGTKVRAGGMVTYVPYSMGRMEYNWGPDAASFRPERWI-NEDGAFRNASPFKFTA

Sorghum LVYLHACVTETLRLYPAVPQDPKGILEDDVLPDGTKVRAGGMVTYVPYSMGRMEYNWGPDAASFRPERWI-NEEGAFRNASPFKFTA

Rice LVYLHACVTETLRLYPAVPQDPKGIVEDDVLPDGTKVRAGGMVTYVPYSMGRMEYNWGPDAASFRPERWLSGDGGAFRNASPFKFTA

FIELDER_A_GENOME_2 LVYLHACVTETLRLYPAVPQDPKGIAEDDVLPDGTKVRAGGMVTYVPYSMGRMEYNWGPDAASFRPERWI-GDDGAFRNASPFKFTA

FIELDER_B_GENOME_3 LVYLHACVTETLRLYPAVPQDPKGIAEDDVLPDGTKVRAGGMVTYVPYSMGRMEYNWGPDAASFRPERWI-GDDGAFRNASPFKFTA

FIELDER_D_GENOME_1 LVYLHACVTETLRLYPAVPQDPKGIAEDDVLPDGTKVRAGGMVTYVPYSMGRMEYNWGPDAASFRPERWI-GDDGAFRNASPFKFTA

Maize FQAGPRICLGKDSAYLQMKMALAILFRFYSFRLLEGHPVQYRMMTILSM

Sorghum FQAGPRICLGKDSAYLQMKMALAILFRFYSFQLLEGHPVQYRMMTILSM

Rice FQAGPRICLGKDSAYLQMKMALAILFRFYTFDLVEDHPVKYRMMTILSM

FIELDER_A_GENOME_2 FQAGPRICLGKDSAYLQMKMALAILCRFFRFELVEGHPVKYRMMTILSM

FIELDER_B_GENOME_3 FQAGPRICLGKDSAYLQMKMALAILCRFFRFELVEGHPVKYRMMTILSM

FIELDER_D_GENOME_1 FQAGPRICLGKDSAYLQMKMALAILCRFFRFELVEGHPVKYRMMTILSM

**Figure S5** Predicted protein translation products of amplicons derived from UNIMS26 primers. Boxed region denotes amino acid sequence diversity, while highlighted text identifies region corresponding to the Ems26+ target site in maize. Sequence differences in sorghum, rice, and wheat Fielder A-, B-, and D-genomes as compared to maize Ms26 protein are indicated as underlined amino acids. A hyphen (‘-‘) indicates a gap in sequence.


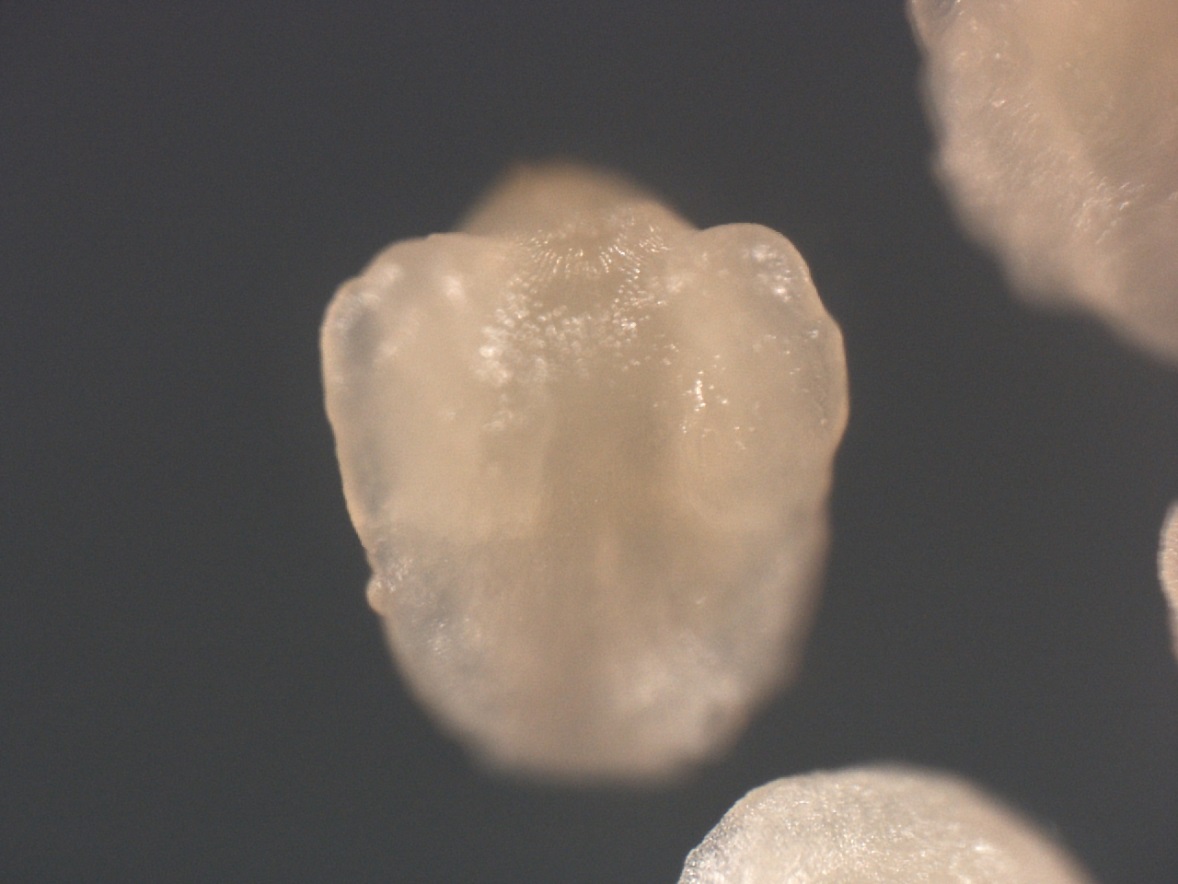


(a)


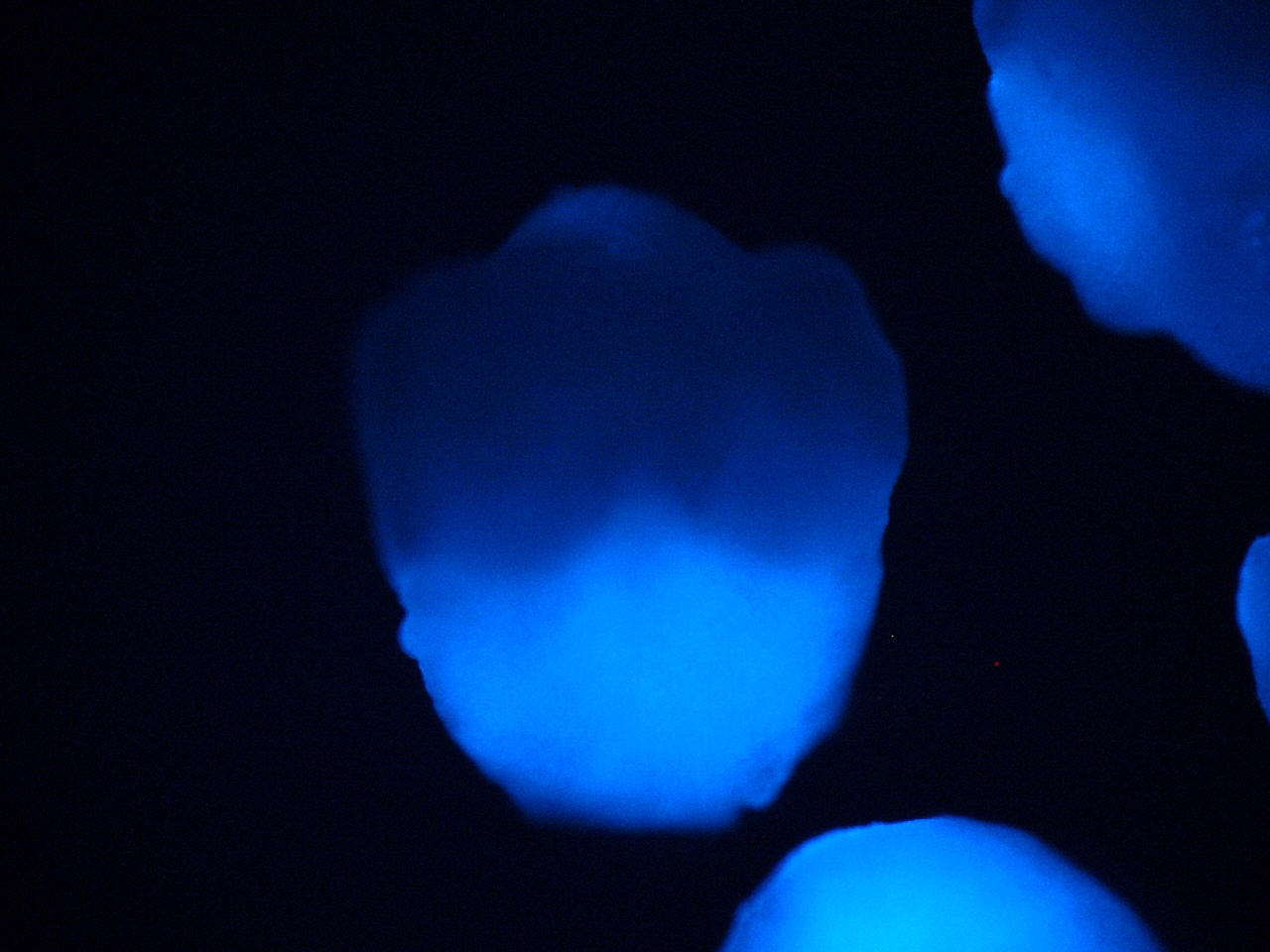


(b)


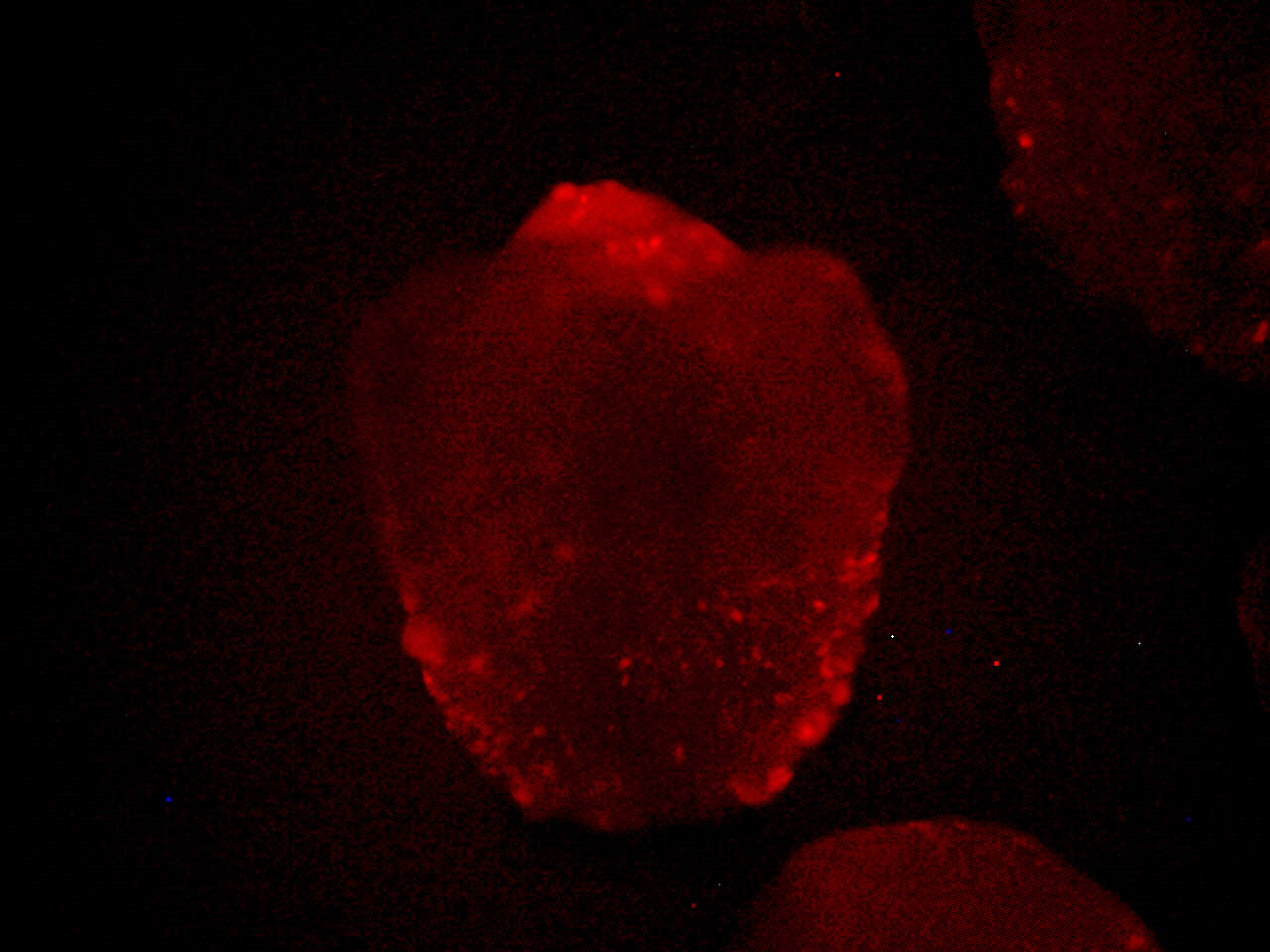


(c)

**Figure S6** Harvested Fielder wheat embryo containing Ems26+ under transcriptional control of a maize temperature-regulated promoter. Wheat embryos containing stably integrated ZmMDHpro:Ems26+ (shown in Figure 4) captured under (a) white light, (b) blue filter, and (c) red filter after treatment at 37ᵒC for 24 hours. Images captured 5 days after treatment. ZmMDHpro, Maize Mannitol dehydrogenase promoter; Ems26+, homing endonuclease targeting Ms26 target site

P D G T K V R A G G M V T Y V P Y S M G R M E Y N W G

CCCGGACGGCACCAAGGTGCGCGCCGGCGGGATGGTGACGTACGTGCCCTACTCCATGGGGCGGATGGAGTACAACTGGGG **Mutant read count**

GTGCGCGCCGGCGGGATGGTGACGTAC 11405

GTGCCCTAC 7683

GGATGGTGACGTACGTGCCCTACTCCATGGGGC 6281

GTAC 5251

GGTGCGCGCCGGCGGGATGGTGACGTA 4879

GTGCCCTACT 4803

GGTGACGTACGTGCCCT 2788

GTGCGCGCCGGCGGGATGGTGAC 2622

GTGCCC 2048

GTGACGTACGTGCCCTAC 1041

**Figure S7** Top 10 amplicon sequences containing mutations from genomic DNA derived from Fielder wheat callus transformed with ZmMDHpro:Ems26+. Ems26+ recognition sequence is highlighted in grey and yellow. The latter indicates the four base-pair overhang, 5’-GTAC-3’, produced by nuclease cutting and is highlighted throughout for orientation purposes. For each mutant, nucleotides shown are deleted sequence. Mutant read counts are listed to the right of each sequence. Amino acid sequence corresponding to the *Ms26* gene is provided as single letters above the WT nucleotide reference sequence.

**Text S1** 4.194 kb region containing wheat *Ms26* ortholog IWGSC 4AS. Sense strand is shown with coding regions highlighted in yellow.

1 CCCTTTCTCC TTCCTCCCTC CTCTCTTTCC CCGGCCGGTG GCGCGCAACC CCGGAGCAAG CAGCGGCGGC AGCGCAGAAC ACCAGAGCAA GAAGCGGAGC

101 ACAGCACGAG CACAAGCAGC GGCGTGCAGC TCGGACGCAT CACCGGAGCA AGCAGGGACC AACGATGGTG AGCTCCTCCG CTGCCTGTGC GTGGCGTGGA

201 CAGCAGCACA TGAGCGCGAG GCCATGGCCG AGCAACCGCG CGGCGGCGCA TGGGCCCGAC CCCACCGGCA CCATCGAGGA GCTCTCCGCA GGGACCTCCT

301 CCACTGCGCG GCCGTCTGCG CCGACGAGGT CGGCCTCGCC GGGAACGCCT CCTCCTCTCC TCTCTCTGGC AGTTGTGCAA AAGAGGGGTT GATTGCTTTT

401 TCTTTTTAGA TCTAAGGGTG TGTGTGTAAA TGTTATGCCA AGTCAGCTCC TTACAATCTG GTCCCACTTG TCAGAAAGTG ATCAAATGAG CCAAATCACC

501 TGATCAGTGC GATTTGCAAA ATGTTTACCG AATCCGCGGT GATTTTTGCA AAAAATTAGT GACCTGGTGG TTTTCCGTAG ATGAAGCCCC AAATGTGGTG

601 GTTTTTCGCA ATTCACTCCA TTTATCAAGT GATGTTCTTC AAAGCAATGG ATTAGGAATG AAATTGTGCC TTACACATAC TCCAATTGTG CTCAACTTGA

701 TAATCTTAGC AATGTTGGGC TATTCATTCA GTGTTTGGCA TTGAGACATG TTTGTCATGA ACTCCCACTG TATACATGTC ATTTATCAAG TGATGATAAT

801 CTTTAAAGTA CATTGGACTG GGATTTAGGA GTGAACTCTG CCCTATACAT AATTCTCAGC TGATAGAGAT CAATTGTGAT CAACTTGAAA GGTGCAGGAA

901 TACACTGTTC GGTTATTCTT TCATTGTTTG CCATGGAGAC ATGAATTATC TTCATCTGAT ACTTGTCAAA TTAGAAGTGA TCTTCATTAG TTGTACATCA

1001 TCCACAGCTG CTACGGTAGT ACCAACAGAA AACGGCCAGC TTGAAAGTTT TGGAATGCAA TGTTAGGGAG TACTATTTAG TAGAGCATAT ATATGATAAA

1101 AGTGTTCTCA TCTGTGGAAC ATATTTATTT GGCAGCACTA GATGCCTCGG CATATTGCAA GGTTTTTAAT ATTTGCGATC TTTTCTGTTT CAAGCTTCTA

1201 ATAAATAGAA GGTGACCACT TTCATCAAAA TTTTCTTCTG TTTAGCTTCT GCTACAAATT TCTAATAAAT ATAGAAGGGG GAACTTTCAG CAAGATTTTT

1301 TATATTTGTG ATTTTCAGGC TTTTTCCATT TAGGGAGAAC ATCAGAGCAC CCCTTGACAG TTGACACCCC TTCATTCGAA ATTTCTCAAC TTGTTCTGCT

1401 TTGACTTCAA AAACTGTTTC ACTGAAAGAT GCACTTTGTA TTGGTTAGTG CGGGTTCAAT AAAGACCAGA TGGACCATAA CCATGGCTCC ATGGCTCCAA

1501 CTGTGAAGAT GACATAATCA CAACGCTAAC TGTCATCAAA CGCATCACCT ACATCCCCCG CAAAACGAAA TAAAAATGCA TCAGTGCATC ACCTACATTT

1601 ATAGTAAAAC AGAAGGAAAA TGCAGAATCC ATGACCTAGC TTAGCACCAA GCACATACTA ACATACCTAG TTATGCATAT AAAAATGAGT GTTTTCTTGG

1701 TCAGCAGATC ACAAAAAGGA CACAAACGGT AGGTTCCATC TAGTCAGGGG GTTAGGTTAG GGACGCCATG TGGATGAGGC AATCTTAATT CTCGGCCACA

1801 CCAAGATTGT TTGGTGCTCG GCGCCACTAA TGCCCAATAT ATTACCTAAC CGAGCCATCC AAATGCTACA TAGAATTAAT CCTCCTGTAG ACTGAACCCA

1901 CTTGATGAGC AGCCCCATGG AGGAAGCTCA CCATGGCATG CCGTCGACGA CGACGGCGTT CTTCCCGCTG GCAGGGCTCC ACAAGTTCAT GGCCATCTTC

2001 CTCGTGTTCC TCTCGTGGAT CTTGGTCCAC TGGTGGAGCC TGAGGAAGCA GAAGGGGCCG AGGTCATGGC CGGTCATCGG CGCGACGCTG GAGCAGCTGA

2101 GGAACTACTA CCGGATGCAC GACTGGCTCG TGGAGTACCT GTCCAAGCAC CGGACGGTCA CCGTCGACAT GCCCTTCACC TCCTACACCT ACATCGCCGA

2201 CCCGGTGAAC GTCGAGCATG TGCTCAAGAC CAACTTCAAC AATTACCCCA AGGTGAAACT GAAAGAACCC CTCAGCCTTG TGAATTTTTT TGCCAAGGTT

2301 CAGAAGTTTA CACTGACACA AATGTCTGAA ATTGTACGTG TAGGGGGAGG TGTACAGGTC CTACATGGAC GTGCTGCTCG GCGACGGCAT CTTCAACGCC

2401 GACGGCGAGC TCTGGAGGAA GCAGAGGAAG ACGGCGAGCT TCGAGTTCGC TTCCAAGAAC CTGAGAGACT TTAGCACGAT CGTGTTCAGG GAGTACTCCC

2501 TGAAGCTGCG CAGCATCCTG AGCCAGGCTT GCAAGGCCGG CAAAGTCGTG GACATGCAGG TAACCGAACT CAGTCCCTTG GTCATCTGAA CATTGATTTC

2601 TTGGACAAAA TTTCAAGATT CTGACGCGAG CGAGCGAATT CAGGAGCTGT ACATGAGGAT GACGCTGGAC TCGATCTGCA AGGTGGGGTT CGGGGTCGAG

2701 ATCGGCACGC TGTCGCCGGA GCTGCCGGAG AACAGCTTCG CGCAGGCGTT CGACGCCGCC AACATCATCG TGACGCTGCG GTTCATCGAC CCGCTGTGGC

2801 GCGTGAAGAA GTTCCTGCAC GTCGGCTCGG AGGCGCTGCT GGAGCAGAGC ATCAAGCTCG TCGACGAGTT CACCTACAGC GTCATCCGCC GGCGCAAGGC

2901 CGAGATCGTG CAGGCCCGGG CCAGCGGCAA GCAGGAGAAG GTGCGTACGT GATCGTCGTC GTCAAGCTCC GGATCGCTGG TTTGTGTAGG TGCCATTGAT

3001 CACTGACACA CTAGCTGGGT GCGCAGATCA AGCACGACAT ACTGTCGCGG TTCATCGAGC TGGGCGAGGC CGGCGGGGAC GACGGCGGCA GCCTGTTCGG

3101 GGACGACAAG GGCCTCCGCG ACGTGGTGCT CAACTTCGTG ATCGCCGGGC GGGACACCAC GGCCACGACG CTCTCCTGGT TCACCTACAT GGCCATGACG

3201 CACCCGGCCG TGGCCGAGAA GCTCCGCCGC GAGCTGGCCG CCTTCGAGGC GGACCGCGCC CGCGAGGATG GCGTCGCGCT GGTCCCCTGC AGCGACTCAG

3301 ACGGCGACGG CTCCGACGAG GCCTTCGCCG CCCGCGTGGC GCAGTTCGCG GGGCTGCTGA GCTACGACGG GCTCGGGAAG CTGGTGTACC TCCACGCGTG

3401 CGTGACGGAG ACGCTGCGCC TGTACCCGGC GGTGCCGCAG GACCCCAAGG GCATCGCGGA GGACGACGTG CTCCCGGACG GCACCAAGGT GCGCGCCGGC

3501 GGGATGGTGA CGTACGTGCC CTACTCCATG GGGCGGATGG AGTACAACTG GGGCCCCGAC GCCGCCAGCT TCCGGCCGGA GCGGTGGATC GGCGACGACG

3601 GCGCGTTCCG CAACGCGTCG CCGTTCAAGT TCACGGCGTT CCAGGCGGGG CCGCGGATCT GCCTCGGCAA GGACTCGGCG TACCTGCAGA TGAAGATGGC

3701 GCTGGCCATC CTGTGCAGGT TCTTCAGGTT CGAGCTCGTG GAGGGCCACC CCGTCAAGTA CCGCATGATG ACCATCCTCT CCATGGCGCA CGGCCTCAAG

3801 GTCCGCGTCT CCAGGGCGCC GCTCGCCTGA TCTTGACCTG GTTCCGGCGA CGGTGATGGA CGCTCCGGTG GCTGGCTGGC CGGACGGCCG GCGCGTTATG

3901 ACAGGCTCGA TTTAGCTTGG CAACTGTGAT AAACTCGTAT ATGTAGGCAG AGTGGAGAGG GTGTTGATCG ATTCGCCATG GACGTTGCTC GTCCGTTGTT

4001 ACCATCGTAC CATGTTTGTA TTGCTTCTAG ATCACTTTAT AGTTCGTGTT TGTTCTTGAG CCTAAGTATT TATTGCACAT TTCAAAAGTG ACAAATGTAT

4101 GCAATTGTCT TTTTGGGGTG TTTTCTAAGG GTAGTATTTT CGTAGATTTA TTTTGTCGAC CAAACCCTGG CCGTCACACA TGATTCGATC CCTC

**Text S2** Spacer sequence (136 bp) containing multiple nuclease target sites. Ems26+ recognition site (yellow highlight), I-SceI (red font), I-CRE (green highlight), and Lig34 (gray highlight)

gctagcatctgatggtgacgtacgtgccctactcgatggggctagggataacagggtaatactgaagctactcaaaacgtcgtgagacagtttgcggaggatatatatacctcacacgtacgcgtagttcgctagc

**Text S3** Nucleotide sequence of Ems26+ target site (underlined) present on Chromosome 7 in *S. bicolor* Tx430.

caaaaatgaaccgtcacagatgatagaattctatgaccatatcttaaaacgtcacatgtaaaccgtcatgtataaggatttttcttgtagtgacaggtgatcgcgcgagcatgagcacgatctccgggctccggtccgcgatgcaatgcagacgatgtggctgtccacacagcatgcactgagtgaacttgcattgcgtgtaatgcacttggacgcaggaccccaaggggatcctggaggacgacgtgctgccggacgggacgaaggtgagggccggcgggatggtgacgtacgtgccctactcgatggggcggatggagtacacatatgattccctgtgtctcgtgtactacccattttattccctgttcacacatatacgctgatccgtattgccagagtaaatacttcctacactyccctaaactatagataggatcataggagaccatatatgctgctgacgtaaaacaatggcatgaatacaagggcgaattctgcagatatcc

**Text S4** Predicted 550 amino acid sequence encoded by wheat *Ms26* ortholog IWGSC 4AS coding region.

meeahhgmpstttaffplaglhkfmaiflvflswilvhwwslrkqkgprswpvigatleqlrnyyrmhdwlveylskhrtvtvdmpftsytyiadpvnvehvlktnfnnypkgevyrsymdvllgdgifnadgelwrkqrktasfefasknlrdfstivfreyslklrsilsqackagkvvdmqelymrmtldsickvgfgveigtlspelpensfaqafdaaniivtlrfidplwrvkkflhvgsealleqsiklvdeftysvirrrkaeivqarasgkqekikhdilsrfielgeaggddggslfgddkglrdvvlnfviagrdttattlswftymamthpavaeklrrelaafeadraredgvalvpcsdsdgdgsdeafaarvaqfagllsydglgklvylhacvtetlrlypavpqdpkgiaeddvlpdgtkvraggmvtyvpysmgrmeynwgpdaasfrperwigddgafrnaspfkftafqagpriclgkdsaylqmkmalailcrffrfelveghpvkyrmmtilsmahglkvrvsrapla*

**Text S5** Maize-optimized Ems26+ coding region. The amino-terminal nuclear localization and intron sequences are shown as yellow and italicized text, respectively.

atggctccgaagaagaagcgcaaggtccacatgaacaccaagtacaacaaggagttcctcctctacctggcaggtttcgtggacggcgatgggtctatcatcgcccagattgatccgcaacagtcctacaagttcaagcactcgctgcggctgaggttcacggtcactcagaagacgcagcgcaggtggttcctcgataagctggtcgacgaaatcggagtcggcaaggtgcgggacaggggctctgtcagcgactacatcctctgtcagatcaagccgctccacaacttcctgacccagctgcagcccttcctcaagctcaagcagaagcaggccaacctggtgctcaagatcatcgagcagctgccatctgccaaggagtcaccagacaagttccttgag*gtaagtttctgcttctacctttgatatatatataataattatcattaattagtagtaatataatatttcaaatatttttttcaaaataaaagaatgtagtatatagcaattgcttttctgtagtttataagtgtgtatattttaatttataacttttctaatatatgaccaaaacatggtgatgtgcag*gtctgcacctgggtcgatcagatcgctgccctgaacgactccaagacgaggaagaccacctccgagaccgtcagggctgtgctggactcactcccaggatccgttggcggtctcagcccttctcaggctagctcggctgcttcctcagccagcagctcacctggctccggtatcagcgaggctctcagagcaggtgccaccaagtccaaggagttcctcctgtacctggcaggcttcgttgacggcgacggctcgatctgcgcgtccattgacccgaaccagtcgtgtaagttcaagcatcagctgcgcctgcgctttaccgtcacgcagaagacccagaggcgctggttcctggacaaactggtggacgagatcggggtcgggaaggtgtacgacagagggagcgttagcgactaccggctgtgccagatcaagccgctccacaacttcctgacgcagctccaacccttcctgaagctgaagcagaagcaggcgaaccttgtgctgaagatcattgagcagctgccgagcgccaaggagagccctgacaagttcctggaggtctgcacctgggtcgaccagatcgctgccctcaacgactccaagaccaggaagaccacgagcgagaccgttcgggctgtcctggacagcctctccgagaagaagaagtcgagcccgtag

**Data S1** Experimental procedures detailing plant transformation and tissue culture methods.

***Rice***

Rice transformation was conducted as described by [Hervé and Kayano (2006](#_ENREF_5)), and [Toki (1997](#_ENREF_7)) with modifications. Dehusked Kitaake rice seeds were sterilized by immersing in 100% EtOH for 10 min, followed by rocking seeds in 40% bleach + TritonX-100 (a few drops) for 15 min, and repeated without surfactant for 15 min. Seeds were rinsed four times with sterile water and blotted dry on sterile filter paper. Seeds were placed into seed pre-culture media [PCM: N6 salts and vitamins ([Chu, 1975](#_ENREF_3)), 300 mg L^-1^ casein hydrolysate, 2.1 g L^-1^ L-proline, 0.1 g L^-1^ myo-inositol, 2 mg L^-1^ 2,4-Dichlorophenoxyacetic acid, 0.5 mg L^-1^ thiamine, 30 g L^-1^ sucrose, and 3.0 g L^-1^ gelrite, pH 5.8], with only the embryo of the rice seed protruding above the surface of the media, at a density of 20 seeds per 100 mm x 25 mm plate ([Hervé and Kayano, 2006](#_ENREF_5)). Seed were incubated at 32ᵒC in the light for 7-10 days. Young callus from seed was used for biolisitc or *Agrobacterium-*mediated transformation.

*Biolistic transformation of rice scutellum*.

Forceps were used to transfer the scutellum calli onto pre-shooting media [PSM: N6 salts and vitamins ([Chu, 1975](#_ENREF_3)), 2.88 g L^-1^ L-proline, 1 mg L^-1^ 2,4-Dichlorophenoxyacetic acid, 0.5 mg L^-1^ thiamine, 120 g L^-1^ sucrose, 30 g L^-1^ mannitol, 30 g L^-1^ sorbitol, and 6.3 g L^-1^ Sigma agar, pH 5.8]. Scutellum-derived calli from 10 rice seed were placed in a small circle in the center of the plate (60 mm x 25 mm) to facilitate bombardment and incubated at 27ᵒC for 4 hours. For biolistic transformation of plasmids, DNA was precipitated onto 0.6 µm (average diameter) gold microprojectiles using a water-soluble cationic lipid TFX-50 as follows: 50 µl of gold particles (water solution, 10 mg/ml) and 5 µl solution of TFX-50 were added to the premixed DNA constructs (1 µg), mixed gently, and incubated on ice for 10 min. DNA-coated gold particles were then centrifuged at 10,000 rpm for 1 min. The pellet was rinsed with 100 µl of absolute alcohol and resuspended by a brief sonication. Immediately after sonication, DNA-coated gold particles were loaded onto the center of a macrocarrier (10 µl each) and allowed to air dry. One hundred ng of DNA were introduced into rice scutellum using a PDS-1000/He Gun (Bio-Rad Laboratories, Inc.) with a rupture pressure of 650 PSI. Bombarded callus was then placed in the dark at 27ᵒC overnight. Callus was transferred to PCM at a density of 20 calli per plate for 1 week in the light at 32ᵒC then transferred to PCM containing 400 mg L^-1^ carbenicillin and 2 mg L^-1^ glufosinate. Callus growing in the presence of glufosinate was subcultured for approximately 4 weeks at 32ᵒC in continuous light. Embryogenic calli were placed onto Regeneration Medium [MS salts and vitamins ([Murashige and Skoog, 1962](#_ENREF_6)), 2 g L^-1^ casein hydrolysate, 30 g L^-1^ sucrose, 40 g L^-1^ sorbitol, 100 mg L^-1^ myo-inositol, 0.02 mg L^-1^ NAA (1-Naphthaleneacetic acid)-proline, 2 mg L^-1^ kinetin, and 3.0 g L^-1^ gelrite, pH 5.8] for 2 weeks under continuous light at 32ᵒC. Plantlets arising from calli were transferred on to rooting media [MS salts and vitamins ([Murashige and Skoog, 1962](#_ENREF_6)), 2 g L^-1^ casein hydrolysate, 40 g L^‑1^ sucrose, 100 mg L^-1^ myo-inositol, 1.5 g L^-1^ gelrite, pH 5.6] under continuous light to promote shoot and root development. After 2 weeks, rice plants were transferred to soil in flats under a humidity dome for 1 week then transferred to pots and grown under greenhouse conditions.

*Agrobacterium-mediated transformation of rice*.

An overnight 25 ml culture (28ᵒC shaking at 250-300 RPM) of LBA4404 harboring the a binary vector was grown in Agro culture medium (10.5 g L^-1^ K_2_HPO_4_, 4.5 g L^-1^ KH_2_PO_4,_ 1 g L^-1^ NH4Cl, 0.5 g L^-1^ C_6_H_5_O_7_•2H_2_O•3Na, 2 g L^-1^ sucrose, 0.12 g L^-1^ MgSO_4,_ 50 mg L^-1^ spectinomycin, and 100μM acetosyringone) to a density of < 1 at OD_550_. Cells were pelleted at 6000X g for 5 min and gently resuspended in Agro resuspension media [N6 salts and vitamins ([Chu, 1975](#_ENREF_3)), 0.69 g L^-1^ L-proline, 0.5 mg L^-1^ thiamine, 1.5 mg L^-1^ 2,4‑Dichlorophenoxyacetic acid, 68.5 g L^-1^ sucrose, 36 g L^-1^ glucose, and 100 μM acetosyringone, pH 5.2] to obtain an OD_550_ of < 0.1. Sterilized pre-cultured scutellum-derived calli were incubated with the *Agrobacterium* culture in 50 ml conical culture tubes sufficient to cover seeds and mixed occasionally for 5-10 min at room temperature. Infected calli from scutellum were blotted dry on sterile filter paper and co-cultivated by placing seeds on a pre-moistened filter paper disk with 0.5 ml of Agro re-suspension medium on a 100 x 25 mm Agro co-cultivation medium plate (N6 salts and vitamins, 0.69 g L^-1^ L-proline, 0.5 mg L^-1^ thiamine, 2.0 mg L^-1^ 2,4-Dichlorophenoxyacetic acid, 30 g L^-1^ sucrose, 10 g L^-1^ glucose, 300 mg L^-1^ casein hydrolysate, 0.1 g L^-1^ myo-inositol, and 100 μM acetosyringone, pH 5.2) and incubated in the dark at 21ᵒC for 72 hours. Co-cultivation was terminated by washing seeds gently in sterile water containing 400 mg L^-1^ carbinicillin, 100 mg L^-1^ vancomycin, and 100 mg L^-1^ Timentin, repeating until clear. Infected scutellum sections were placed onto Agro maintenance medium (N6 salts and vitamins, 0.5 mg L^-1^ thiamine, 2.0 mg L^-1^ 2,4‑Dichlorophenoxyacetic acid, 30 g L^-1^ sucrose, 300 mg L^-1^ casein hydrolysate, 0.1 g L^-1^ myo-inositol, and 100 mg L^-1^ carbinicillin, pH 5.8). Transient fluorescing calli, identified using a Leica M165 FC fluorescence microscope, were visible within 2-4 days after the termination of the co-culture. Growing fluorescent callus sectors were transferred to fresh Agro maintenance medium, repeating the transfer again after 10 additional days (20 days total after termination of co-cultivation), carefully selecting for fluorescent tissues. Uniformly fluorescing calli (1 cm^3^ in size) were used for plant regeneration as described above.

***Sorghum transformation***

Sorghum immature embryos (Tx430) were transformed using the Agrobacterium tumefaciens strain LBA4404 harboring a PSB11 backbone binary vector as previously described by [Zhao *et al.* (2000](#_ENREF_8)) with the exception that glufosinate was omitted during tissue culture process. Fluorescing growing calli were identified using a Leica M165 FC fluorescence microscope, subcultured and selected for plant regeneration.

***Wheat transformation***

Wheat (*Triticum aestivum* L., cv. Fielder) plants were grown in a growth chamber or greenhouse. Immature seeds with immature embryos (IEs) of about 1.5 to 2.5 mm collected from spikes were surface-sterilized for 20 min in 15% (v/v) bleach (5.25% sodium hypochlorite) plus one drop of Tween 20 followed by three washes in sterile water. IEs were isolated and placed in 1.0 ml of liquid infection medium with 0.25 mL of autoclaved sand in 2 mL microcentrifuge tubes. The IEs were treated with centrifuging in the infection medium and then inoculated with *Agrobacterium*. The suspension of *Agrobacterium* and IEs was poured onto co-cultivation medium ([Zhao *et al.*, 2000](#_ENREF_8)). The IEs were placed embryo axis side down on the media, and incubated in the dark at 21ᵒC. After 3 days IEs were transferred to DBC4 medium containing 100 mg/L cefotaxime (PhytoTechnology Laboratories) and then incubated at 26-28ᵒC in low light for 2 weeks. DBC4 medium is DBC3 green regenerative medium ([Cho *et al.*, 1998](#_ENREF_1)) modified with 1.0 mg L^-1^ of 6-benzylaminopurine (BAP). The tissues were then were transferred to DBC6 medium (a modified DBC3 medium with 0.5 mg L^-1^ 2,4-dichlorophenoxyacetic acid and 2.0 mg L^-1^ BAP) containing 150 mg L^-1^ cefotaxime for another 2 weeks. Fluorescing callus sectors were identified using a Leica M165 FC fluorescence microscope, cut from the non-transformed tissues, and placed on MSA regeneration medium [MSB ([Cho *et al.*, 2014](#_ENREF_2)) without Indole-3-butyric acid] with 150 mg L^-1^ cefotaxime. After sectors have developed into small plantlets, they were transferred to MSB rooting medium. During each transfer, plantlets were checked for DsRED gene expression and any non-expressing or chimeric tissues were removed. Plants were transferred to pots in the greenhouse for phenotypic and molecular studies.

Cho, M.-J., Jiang, W. and Lemaux, P.G. (1998) Transformation of recalcitrant barley cultivars through improvement of regenerability and decreased albinism. *Plant Science* **138**, 229-244.

Cho, M.J., Wu, E., Kwan, J., Yu, M., Banh, J., Linn, W., Anand, A., Li, Z., TeRonde, S., Register, J.C., 3rd, Jones, T.J. and Zhao, Z.Y. (2014) *Agrobacterium*-mediated high-frequency transformation of an elite commercial maize (*Zea mays* L.) inbred line. *Plant Cell Rep* **33**, 1767-1777.

Chu, C.C. (1975) Establishment of an efficient medium for anther culture of rice through comparitive experiments on the nitrogen sources. *Scientia Sinica* **18**, 659-668.

Djukanovic, V., Smith, J., Lowe, K., Yang, M., Gao, H., Jones, S., Nicholson, M.G., West, A., Lape, J., Bidney, D., Falco, C.S., Jantz, D. and Lyznik, L.A. (2013) Male-sterile maize plants produced by targeted mutagenesis of the cytochrome P450-like gene (*MS26*) using a re-designed I–*Cre*I homing endonuclease. *The Plant Journal* **76**, 888-899.

Hervé, P. and Kayano, T. (2006) Japonica Rice Varieties (*Oryza sativa*, Nipponbare, and Others). In: *Agrobacterium Protocols* (Wang, K. ed) pp. 213-222. Humana Press.

Murashige, T. and Skoog, F. (1962) A revised medium for rapid growth and bioassays with tobacco tissue cultures. *Physiol Plant* **15**, 473 - 497.

Toki, S. (1997) Rapid and efficient Agrobacterium -mediated transformation in rice. *Plant Molecular Biology Reporter* **15**, 16-21.

Zhao, Z.-Y., Cai, T., Tagliani, L., Miller, M., Wang, N., Pang, H., Rudert, M., Schroeder, S., Hondred, D., Seltzer, J. and Pierce, D. (2000) *Agrobacterium*-mediated sorghum transformation. *Plant Molecular Biology* **44**, 789 - 798.
